# Elevated PDGF‐BB from Bone Impairs Hippocampal Vasculature by Inducing PDGFR*β* Shedding from Pericytes

**DOI:** 10.1002/advs.202206938

**Published:** 2023-04-27

**Authors:** Guanqiao Liu, Jiekang Wang, Zhiliang Wei, Ching‐Lien Fang, Ke Shen, Cheng Qian, Cheng Qi, Tong Li, Peisong Gao, Philip C. Wong, Hanzhang Lu, Xu Cao, Mei Wan

**Affiliations:** ^1^ Department of Orthopaedic Surgery Johns Hopkins University School of Medicine Ross Building, Room 232, 720 Rutland Avenue Baltimore MD 21205 USA; ^2^ The Russell H. Morgan Department of Radiology and Radiological Science The Johns Hopkins University School of Medicine Baltimore MD 21205 USA; ^3^ Department of Pathology Johns Hopkins University School of Medicine Baltimore MD 21205 USA; ^4^ Division of Allergy and Clinical Immunology Johns Hopkins University School of Medicine Baltimore MD 21224 USA; ^5^ Department of Neuroscience Johns Hopkins University School of Medicine Baltimore MD 21205 USA

**Keywords:** blood‐brain barrier permeability, bone, circulating platelet‐derived growth factor‐BB, PDGFR*β*, receptor shedding

## Abstract

Evidence suggests a unique association between bone aging and neurodegenerative/cerebrovascular disorders. However, the mechanisms underlying bone‐brain interplay remain elusive. Here platelet‐derived growth factor‐BB (PDGF‐BB) produced by preosteoclasts in bone is reported to promote age‐associated hippocampal vascular impairment. Aberrantly elevated circulating PDGF‐BB in aged mice and high‐fat diet (HFD)‐challenged mice correlates with capillary reduction, pericyte loss, and increased blood‐brain barrier (BBB) permeability in their hippocampus. Preosteoclast‐specific *Pdgfb* transgenic mice with markedly high plasma PDGF‐BB concentration faithfully recapitulate the age‐associated hippocampal BBB impairment and cognitive decline. Conversely, preosteoclast‐specific *Pdgfb* knockout mice have attenuated hippocampal BBB impairment in aged mice or HFD‐challenged mice. Persistent exposure of brain pericytes to high concentrations of PDGF‐BB upregulates matrix metalloproteinase 14 (MMP14), which promotes ectodomain shedding of PDGF receptor *β* (PDGFR*β*) from pericyte surface. MMP inhibitor treatment alleviates hippocampal pericyte loss and capillary reduction in the conditional *Pdgfb* transgenic mice and antagonizes BBB leakage in aged mice. The findings establish the role of bone‐derived PDGF‐BB in mediating hippocampal BBB disruption and identify the ligand‐induced PDGFR*β* shedding as a feedback mechanism for age‐associated PDGFR*β* downregulation and the consequent pericyte loss.

## Graphical abstract

The study establishes the role of platelet‐derived growth factor‐BB (PDGF‐BB) as a new member of the bone‐derived pro‐aging factor family. Once PDGF‐BB reaches levels beyond the physiological range during aging or under pathological conditions, it induces the shedding of its receptor PDGF receptor *β* (PDGFR*β*) from cell membrane of the brain pericytes through matrix metalloproteinase 14 (MMP14), leading to pericyte loss and cerebrovascular impairment.

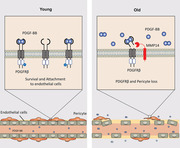

## 1 Introduction

Skeletal aging has a unique connection to cerebrovascular disease and brain aging. Osteoporosis, a common age‐associated bone disorder, is more prevalent in people with neurodegenerative conditions, and patients with dementia have a much higher risk of experiencing low bone mineral density (BMD)^[^ ^1^ ^]^ than age‐matched neurotypical (i.e., those without dementia) adults. Retrospective cross‐sectional studies show that low BMD is independently associated with white matter diseases, characterized by pathological changes in small blood vessels and capillaries of the brain.^[^ ^2^ ^]^ Although the concept of a bone‐brain axis has been proposed,^[^ ^3^ ^]^ little is known about the underlying cellular and molecular basis and how this axis involves in the aging process of cerebrovascular system.

Highly coordinated neurovascular interaction is vital for maintaining the homeostasis of the brain. A defining feature of brain vasculature is a physical blood‐brain barrier (BBB), which functions as a module within the greater context of a multicellular neurovascular unit. BBB, formed by microvascular endothelial cells, pericytes, and astrocytes, restricts entry of blood‐derived neurotoxic molecules into the brain from peripheral circulation and regulates the transport of molecules into and out of the brain to maintain tightly controlled neuronal milieu for proper neuronal functioning.^[^ ^4^ ^]^ Pericytes, which ensheath the brain capillary wall and share the same basement membrane with capillary endothelial cells, are a key cell type for maintaining BBB integrity.^[^ ^5^ ^]^ Accumulating human and mouse studies have shown that brain capillary pericyte dysfunction and/or deficiency leads to BBB breakdown, which in turn may contribute to the pathogenesis of many neurological disorders such as Alzheimer's disease (AD), stroke, traumatic brain injury, diabetes, and amyotrophic lateral sclerosis (ALS).^[^ ^6^ ^]^

The platelet‐derived growth factor‐BB (PDGF‐BB)/PDGF receptor β (PDGFR*β)* signaling‐mediated pericyte‐endothelial crosstalk plays a crucial role in the establishment and maintenance of the pericyte coverage on cerebral microvasculature and the BBB homeostasis during embryonic development^[^ ^7^ ^]^ and adulthood.^[^ ^8^ ^]^ Endothelial cells of the brain are enriched for and secrete PDGF‐BB which binds to its receptor PDGFR*β* on pericytes and activates intracellular signaling pathways that promote pericyte migration, proliferation, and attachment to endothelial cells to support endothelial tube formation and stabilization.^[^ ^9^ ^]^ Deletion of *Pdgfb* or *Pdgfrb* genes in mice results in vascular defect‐related embryonic lethality due to a lack of pericytes.^[^ ^7^, ^10^ ^]^ The role of the PDGF‐BB/PDGFR*β* signaling in postnatal BBB has been studied mostly from the *Pdgfb* or *Pdgfrb* mutant mice, such as heterozygous *Pdgfb* and *Pdgfrb* null mutants, *Pdgfb* ^ret/ret^ mice with deletion of the retention motif of PDGF‐B causing diminished PDGF‐BB bioavailability, and *Pdgfrb* ^F7/F7^ mice with reduced signaling competence on the receptor side.^[^ ^7^, ^10^, ^11^ ^]^ These mutant mice exhibit more or less pericyte loss, dilation of micro‐vessels, BBB impairment^[^ ^7^, ^12^ ^]^ as well as the formation of microvascular calcification,^[^ ^13^ ^]^ suggesting an important role of PDGF‐BB/PDGFR*β* signaling in pericyte and BBB biology.

During normal aging, morphological/structural changes of the cerebral microvasculature have been noticed from human and animal studies.^[^ ^14^ ^]^ These changes include reduced capillary density and diameter, tight junction protein number and length, pericyte number and vessel coverage, astrocyte end‐feet coverage, neuroinflammation, and increased basement membrane thickness.^[^ ^15^ ^]^ Functionally, there is increased BBB permeability, decreased cerebral blood flow in brain regions involved in cognition,^[^ ^16^ ^]^ and changed BBB transcytosis with age.^[^ ^15^, ^17^ ^]^ Pericyte loss and BBB breakdown are early events in the aging brain that begin in the hippocampus.^[^ ^16b^ ^]^ However, as one of the most studied signaling pathways for BBB development and adult homeostasis, there have been very few studies that evaluate the changes of PDGF‐BB/PDGFR*β* signaling during natural aging as well as how it is involved in age‐associated cerebrovascular impairment. Clinical data from patients have shown that soluble‐PDGRF*β* (sPDGRF*β*), as a marker of pericyte degeneration, is elevated in the cerebrospinal fluid (CSF) of patients with Alzheimer's disease and mild cognitive impairment and correlates with imaging‐based assessments of BBB permeability.^[^ ^16^, ^18^ ^]^ The finding supports the involvement of PDGF‐BB/PDGFR*β* signaling in disease pathology and suggests that this important signaling pathway regulating BBB may undergo substantial alteration with advancing age.

We previously demonstrated that tartrated resistant acid phosphatse^+^ (TRAP^+^) preosteoclasts secretes PDGF‐BB,^[^ ^19^ ^]^ and deletion of *Pdgfb* selectively from preosteoclasts results in approximately 40% reduction in serum PDGF‐BB concentration in mice, suggesting skeletal preosteoclast is one of the main sources of PDGF‐BB under normal healthy condition. Most importantly, we recently revealed that TRAP^+^ preosteoclasts are a main cell type contributing to the elevation of serum PDGF‐BB during aging and under metabolic stress.^[^ ^20^ ^]^ Specifically, while aged mice and those fed high‐fat diet (HFD) had higher serum PDGF‐BB relative to young mice and chow diet (CHD) mice respectively, conditional *Pdgfb* knockout mice selectively in TRAP^+^ preosteoclasts (Pdgfb^cKO^) had a normalized serum PDGF‐BB concentration. Moreover, preosteoclast‐specific *Pdgfb* transgenic mice (Pdgfb^cTG^) had a much higher serum PDGF‐BB level relative to their WT littermates. Therefore, skeletal preosteoclast‐derived PDGF‐BB is both sufficient and required to cause circulating PDGF‐BB elevation.^[^ ^20^ ^]^ In the current study, we took advantage of these two genetic mouse models (i.e., Pdgfb^cKO^ and Pdgfb^cTG^) as well as primary human brain pericytes that expose to different concentrations of PDGF‐BB to determine how increased PDGF‐BB is involved in cerebrovascular changes and BBB impairment during natural aging.

## 2 Result

### 2.1 Elevation of Circulating PDGF‐BB Correlates with Hippocampal Pericyte Loss and BBB Impairment

**Figure 1:**
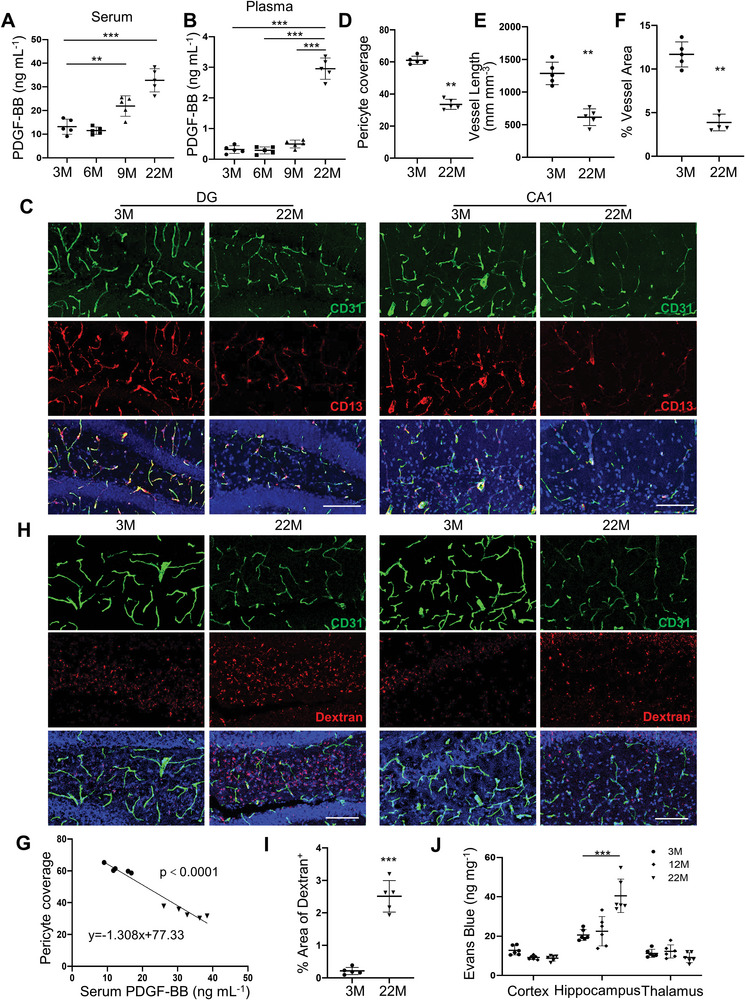
Elevation of circulating PDGF‐BB correlates with hippocampal pericyte loss and BBB impairment. A,B) ELISA analysis of serum (A) and plasma (B) PDGF‐BB concentrations in C57BL/6 mice at 3, 6, 9, and 22 months of age. C) Representative confocal images of CD31 (green) and CD13 (red) double‐immunofluorescence staining in dentate gyrus (DG) (left) and CA1 (right) region of 3‐month‐ and 22‐month‐old mice. DAPI stains nuclei as blue. Scale bar, 100 µm. D) Quantification of CD13^+^ pericyte coverage of the capillaries in hippocampus. E,F) Quantification of percentage of vessel length (E) and vessel area (F) in hippocampus area. n = 5. G) Spearman correlation of serum PDGF‐BB level and pericyte coverage of capillaries in hippocampus in 3‐ and 22‐month‐old mice. H,I) BBB permeability was quantified from the leaking of 10 kDa dextran‐conjugated fluorophores into the DG and CA1 parenchymal space outside the vessels in 3‐ and 22‐month‐old WT mice. Vessels were identified by the CD31(green), while leaks were identified by leakage (red) of fluorescence outside the vessels (H). Scale bar, 100 µm. DAPI stains nuclei as blue. (I) BBB leakage is quantified by the percentage of Dextran^+^ signal area comparing in hippocampus of 3‐ and 22‐month‐old WT mice. n = 5. J) In vivo Evans blue permeability assay in cortex, hippocampus, and thalamus of 3‐, 12‐, and 22‐month‐old mice. Data are shown as the mean ± SD, **p<0.01, ***p<0.001, as determined by unpaired two‐tailed Student's *t* test (for two group comparison) or One‐way ANOVA (for multiple group comparison).

To evaluate the changes in PDGF‐BB/PDGFR*β* signaling during normal aging, we measured serum PDGF‐BB concentration in mice with advancing age. An age‐dependent elevation of serum PDGF‐BB concentration was observed in mice, starting at 9 months of age (**Figure** **1**A). Relative to young (3 months) mice, the serum PDGF‐BB level in aged (22 months) mice is nearly threefold higher. It has been reported that the PDGF‐BB concentration in serum is much higher than that in plasma due to PDGF‐BB being released from platelets during serum preparation.^[^ ^21^ ^]^ Remarkedly, we found a more dramatic age‐associated PDGF‐BB elevation in plasma, with approximately 9.4‐fold increase in aged mice as compared to young mice (Figure 1B), suggesting that the elevated PDGF‐BB in aged mice is mainly derived from a non‐platelet source. We also measured the PDGF‐BB concentration in brain tissue extracts from different brain regions by ELISA. PDGF‐BB was higher only in hippocampus with no detectable differences in cortex and thalamus tissue of aged mice relative to young mice (Figure S1, Supporting Information). Double‐immunofluorescence staining of the brain tissue sections revealed reduced brain capillaries and pericyte coverage in the dentate gryus (DG) and cornu ammonis field 1 (CA1) regions of hippocampus in aged mice as compared with those in young mice (Figure 1C–F). Importantly, there is a strong correlation between hippocampal pericyte loss and elevated levels of serum PDGF‐BB (Figure 1G). The reductions in brain capillaries and pericyte coverage were also detected in the cortex region adjacent to hippocampus but not in thalamus in aged mice relative to young mice (Figure S2A–S2J, Supporting Information).

Loss of pericyte coverage is often accompanied by a change in BBB permeability. The relative fluorescence of different molecular size dextran conjugates in parenchyma following intravenous injection was measured. No significant differences in the BBB permeability between young (3‐month‐old) and aged (18‐month‐old) mice were detected for 40 and 70 kDa fluorophores (data not shown). However, the leakage of the 10 kDa fluorophore was greatly increased in aged mice relative to young mice (Figure 1H,I), indicating an increased BBB permeability to smaller compounds with age. Moreover, the extravasations of Evans Blue and serum albumin were greater in hippocampus of aged mice as compared with young mice (Figure 1J, Figure S2K,L, Supporting Information). We previously found that HFD‐challenge mice also had much higher serum PDGF‐BB concentration relative to CHD mice.^[^ ^20^ ^]^ Similar to age‐associated cerebrovascular phenotype, HFD also causes cerebrovascular impairment^[^ ^22^ ^]^ and PDGF‐BB/PDGFR*β* signaling alteration in the brain.^[^ ^23^ ^]^ We therefore assessed cerebrovascular changes in mice fed HFD. Consistently with our previous finding, elevated concentration of PDGF‐BB was also detected in both serum and plasma of mice fed HFD relative to the mice fed normal CHD, with 4.9‐fold and tenfold increase in serum and plasma, respectively (Figure S3A,S3B, Supporting Information). The density of the capillaries and pericyte coverage in the DG and CA1 regions of hippocampus were significantly reduced in HFD mice relative to CHD mice (Figure S3C,D, Supporting Information). Moreover, the extravasation of serum albumin was absent from the hippocampus of CHD mice but was detectable in the HFD mice (Figure S3E,F, Supporting Information).

### 2.2 Elevated Circulating PDGF‐BB Produced by Preosteoclasts is Sufficient to Induce Hippocampal Microvascular Impairment and Cognitive Decline

**Figure 2:**
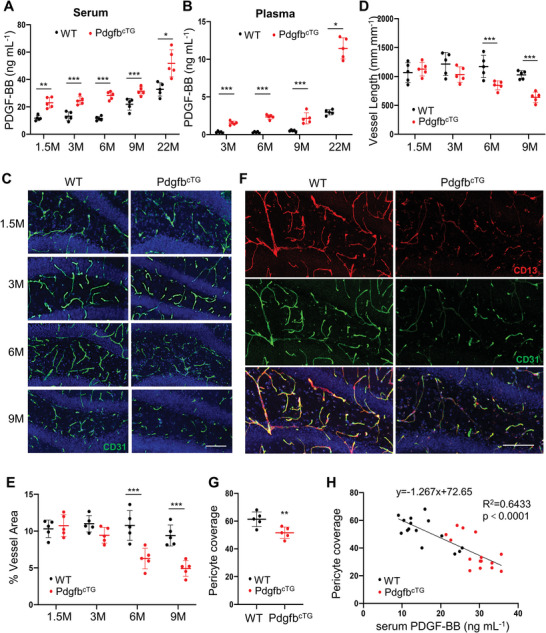
Aberrantly elevated circulating PDGF‐BB is sufficient to induce hippocampal microvascular impairment. A,B) ELISA analysis of serum (A) and plasma (B) PDGF‐BB concentration in Pdgfb^cTG^ mice and WT littermates at 1.5, 3, 6, 9, and 22 months of age. C) Representative confocal images of CD31 (green) immunofluorescence staining in DG region of Pdgfb^cTG^ mice and WT littermates at 1.5, 3, 6, and 9 months of age. DAPI stains nuclei as blue. Scale bar, 100 µm. D,E) Quantification of vessel length (D) and percentage of vessel area (E). F) Representative confocal images of CD31 (green) and CD13 (red) double‐immunofluorescence staining in DG region of 6‐month‐old Pdgfb^cTG^ mice and WT littermates. DAPI stains nuclei as blue. Scale bar, 100 µm. G) Quantification of CD13^+^ pericyte coverage of the capillaries in hippocampus. n = 5. Data are shown as the mean ± SD, *p<0.05, **p<0.01 and ***p<0.001, as determined by unpaired two‐tailed Student's *t* test. H) Spearman correlation of serum PDGF‐BB level and pericyte coverage of capillaries in hippocampus in 3‐, 6‐, and 9‐month‐old Pdgfb^cTG^ mice and WT littermates. n = 15.

We then examined cerebrovascular changes in conditional *Pdgfb* transgenic mice (Pdgfb^cTG^), in which PDGF‐BB is overexpressed in TRAP^+^ preosteoclasts^[^ ^20^, ^24^ ^]^ and results in elevated circulating level of PDGF‐BB.^[^ ^20^ ^]^ Indeed, we found that the serum and plasma concentrations of PDGF‐BB were elevated in Pdgfb^cTG^ mice relative to wild‐type littermates (WT) at all the age groups tested, starting from 1.5 months (**Figure** **2**A,B). Similar to aged mice, PDGF‐BB was higher only in hippocampus of Pdgfb^cTG^ mice relative to WT mice with no detectable differences in cortex and thalamus tissue (Figure S4A–C, Supporting Information). There were decreases in the integrity and density of the hippocampal capillaries in the Pdgfb^cTG^ mice relative to the WT mice at 6 and 9 months of age, as indicated by reduced CD31^+^ blood vessel length and percentage of vessel area in the DG (Figure 2C–E) and CA1 regions (Figure S5, Supporting Information). The brain capillary reduction was not significant in 1.5‐ and 3‐month‐old Pdgfb^cTG^ mice relative to their littermate controls, suggesting that PDGF‐BB elevation‐induced hippocampal vascular change is not an acute response. Similar to the changes in aged mice, CD13^+^ pericytes were markedly reduced in hippocampus of the Pdgfb^cTG^ mice compared with WT mice (Figure 2F). Although the CD31^+^ capillaries were also reduced, decreased percentage of the pericyte coverage of the capillaries was detected in Pdgfb^cTG^ as compared with WT mice (Figure 2G), suggesting a more profound pericyte loss than the blood vessel reduction. Consistent with the finding from aged mice, pericyte loss is strongly correlated with the elevation of serum PDGF‐BB concentration in these mice (Figure 2H). Significant reductions in the density of capillaries were not found in other brain regions, including cortex, thalamus, and hypothalamus in the Pdgfb^cTG^ mice versus WT mice at 6 months of age (Figure S6A–C, Supporting Information). Moreover, unlike aged mice, Pdgfb^cTG^ mice did not exhibit significant pericyte loss in the cortex region that is close to hippocampus (Figure S6D,E, Supporting Information).

**Figure 3:**
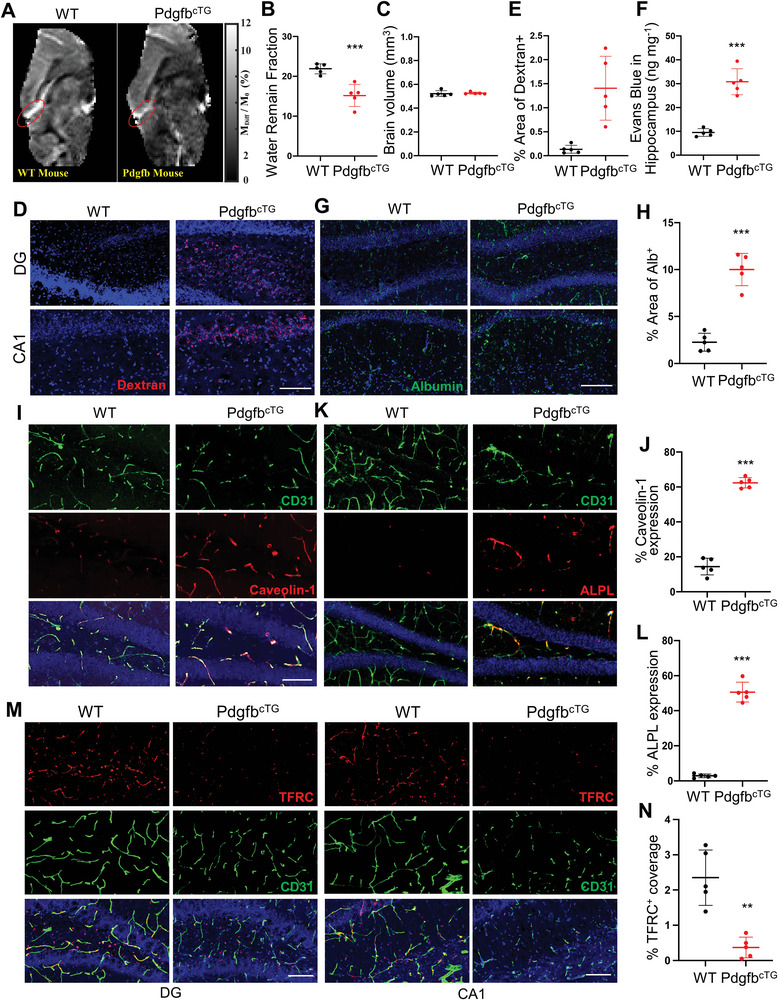
Conditional *Pdgfb* transgenic mice recapitulate aged BBB phenotype. A–C) MRI measurements of 6‐month‐old Pdgfb^cTG^ mice and WT littermates. (A) Presents the WEPCAST images, red ellipsoid shows the calculated vein of mouse brain. (B) Water Remain Fraction and (C) brain volume. The great cerebral vein of Galen was marked with an ellipsoid to show the WEPCAST signal difference between WT and Pdgfb^cTG^ mice. D,E) BBB permeability was quantified from the leaking of 10 kDa dextran‐conjugated fluorophores into the DG and CA1 parenchymal space outside the vessels in 6‐month‐old Pdgfb^cTG^ mice and WT littermates. Vessels were identified by the CD31(green), while leaks were identified by leakage (red) of fluorescence outside the vessels (D). Scale bar, 100 µm. DAPI stains nuclei as blue. (E) BBB leakage is quantified by percentage of Dextran^+^ signal area comparing in hippocampus of 6‐month‐old Pdgfb^cTG^ mice and WT littermates. n = 5. F) In vivo Evans blue permeability assay in hippocampus in 6‐month‐old Pdgfb^cTG^ mice and WT littermates. n = 5. G) Representative immunofluorescence images of DG and CA1 region of hippocampus from Pdgfb^cTG^ mice and WT littermates using antibody against albumin. DAPI stains nuclei blue. Scale bar, 100 µm. H) Quantification of Albumin^+^ signal covered area using Image J. n = 5. I) Representative confocal images of CD31 (green) and Caveolin‐1 (red) double‐immunofluorescence staining in DG region of 6‐month‐old Pdgfb^cTG^ mice and WT littermates. DAPI stains nuclei as blue. Scale bar, 100 µm. J) Quantification of Caveolin‐1 expression of the capillaries in hippocampus. K) Representative confocal images of CD31 (green) and ALPL (red) immunofluorescence staining in DG region of 6‐month‐old Pdgfb^cTG^ mice and WT littermates. DAPI stains nuclei as blue. Scale bar, 100 µm. L) Quantification of ALPL expression of the capillaries in hippocampus. n = 5. M) Representative confocal images of CD31 (green) and TFRC (red) double‐immunofluorescence staining in DG region of 6‐month‐old Pdgfb^cTG^ mice and WT littermates. DAPI stains nuclei as blue. Scale bar, 100 µm. N) Quantification of TFRC expression of the capillaries in hippocampus. Data are shown as the mean ± SD, **p<0.01, ***p<0.001, as determined by unpaired two‐tailed Student's *t* test.

To further elucidate the functional consequence of pericyte loss in vivo, we examined BBB integrity of Pdgfb^cTG^ mice with non‐invasive MRI. Using a WEPCAST MRI technique,^[^ ^16a^ ^]^ we measured BBB's permeability to small molecules such as water and observed that intravascular molecules, in this case non‐invasively labeled water molecules coming from the brain's feeding arteries, were poorly retained when they passed through the capillaries in the Pdgfb^cTG^ mice relative to the WT mice (**Figure** **3**A), suggesting a leaky BBB. Quantitative analyses revealed that there was a significant difference in the water retaining fraction (WRF) between Pdgfb^cTG^ and WT mice (Figure 3B). We also measured brain volume in these mice, and there was not a significant difference in brain volume (Figure 3C), suggesting that functional changes in BBB integrity occurred in the absence of (or preceding) anatomic changes. Furthermore, the leakage of the dextran (Figure 3D,E), extravasations of Evans Blue (Figure 3F), and serum albumin (Figure 3G,H) were greater in the hippocampus of Pdgfb^cTG^ mice compared with those in WT mice. Increased extravasation of Evans Blue was not detected in the cortex of Pdgfb^cTG^ mice versus WT mice (Figure S6F, Supporting Information), indicating a higher BBB permeability specifically in hippocampus.

There is an age‐related shift in plasma protein transport from ligand‐specific receptor‐mediated transcytosis (RMT) to non‐RMT caveolar transcytosis in the aged brain. This shift occurs alongside a loss of RMT‐specific receptor TFRC as well as increases in caveolin‐1 and alkaline phosphatase (ALPL), molecular targets to enhance non‐RMT.^[^ ^15c^ ^]^ Intriguingly, we also detected upregulated expression of non‐RMT markers caveolin‐1 and ALPL in brain capillaries of our Pdgfb^cTG^ mice (Figure 3I–L). Moreover, there was a diminished expression of RMT receptor TFRC in hippocampal capillaries of Pdgfb^cTG^ mice relative to WT mice (Figure 3M,N). The results suggest that Pdgfb^cTG^ mice exhibited an increased non‐specific transcytosis in the cerebral vasculature, faithfully recapitulating the aged cerebrovascular phenotype. To further confirm that bone‐derived PDGF‐BB can be delivered into brain and transmigrates across the BBB into brain tissue, we have generated a new conditional transgenic mouse reporter stain Pdgfb‐tdTom^cTG^, in which tdTomato‐fused PDGF‐BB is expressed in TRAP^+^ preosteoclasts. Our new data shows that tdTom^+^ signaling was not detected in all brain regions in young mice (4‐month‐old), indicating bone‐derived PDGF‐BB could not transmigrate across the BBB into brain tissue in younger mice. Intriguingly, we detected many strong fluorescent tdTom^+^ signals in brain parenchyma outside of the capillaries in 10‐month‐old Pdgfb‐tdTom^cTG^ mice (Figure S7, Supporting Information). The result suggests that persistently increased PDGF‐BB derived from preosteoclasts can penetrate the endothelium to reach other brain cells, such as pericytes.

**Figure 4:**
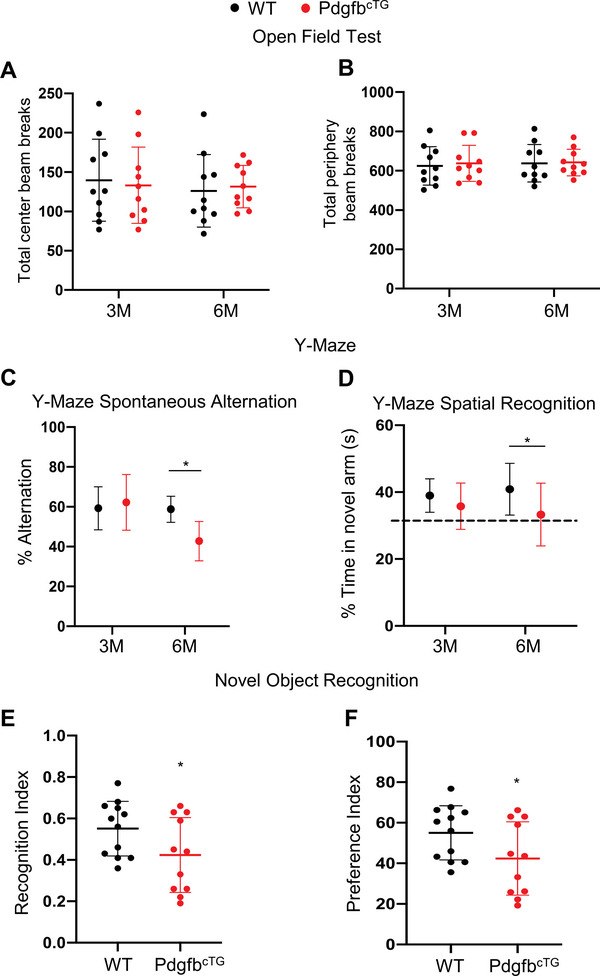
Conditional *Pdgfb* transgenic mice have declined cognitive function. Cognitive mouse behaviors were assessed in 3‐ and 6‐month‐old male Pdgfb^cTG^ mice and WT littermates. A,B) Mice were tested for open‐field, C) spontaneous alternation and D) spatial recognition in Y‐maze, and E,F) novel object recognition. n = 10–12. Data are shown as the mean ± SD, *p<0.05, as determined by unpaired two‐tailed Student's *t* test.

We conducted behavioral tests to determine whether Pdgfb^cTG^ mice have any cognitive impairments. Because capillary changes were mainly detected in the hippocampus of the transgenic lines, we focused on hippocampal‐dependent cognitive tests involving the Y‐maze test and novel object recognition. We previously found that the Pdgfb^cTG^ mice develop osteoarthritis at 5 months of age,^[^ ^24^ ^]^ and for this reason, we avoided physically difficult tests, such as swimming tasks. Measurement of the total center beam breaks (**Figure** **4**A) and total periphery beam breaks in the center (Figure 4B) in an open‐field test revealed there was no statistical difference between the Pdgfb^cTG^ mice and WT mice at 3 and 6 months of age, indicating that any potential differences in the following behavioral tasks would not be due to differences in their general motor function and exploratory activity. We next used the Y‐maze spontaneous alternation and spatial recognition tests, which are the prefrontal cortex and hippocampal‐dependent tasks.^[^ ^25^ ^]^ While control animals at both 3 and 6 months of age display the expected alternation values (≈60%) avoiding the previously visited arms, 6‐month‐old Pdgfb^cTG^ mice alternated at chance (≈40%) level (Figure 4C), indicating impaired working memory. Consistently, the percentage time in the novel arm was also reduced only in the 6‐month‐old Pdgfb^cTG^ mice relative to the age‐matched WT mice (Figure 4D). We then used the novel object recognition test to assess recognition memory.^[^ ^26^ ^]^ In this test, both the recognition index (Figure 4E) and preference index (Figure 4F) were lower in the 6‐month‐old Pdgfb^cTG^ mice as compared with age‐matched WT mice, suggesting recognition memory deficits in transgenic mice.

### 2.3 Normalizing the Circulating PDGF‐BB by Deletion of *Pdgfb* from Preosteoclasts Ameliorates Age‐Associated Hippocampal Microvascular Impairment and Cognitive Decline

**Figure 5:**
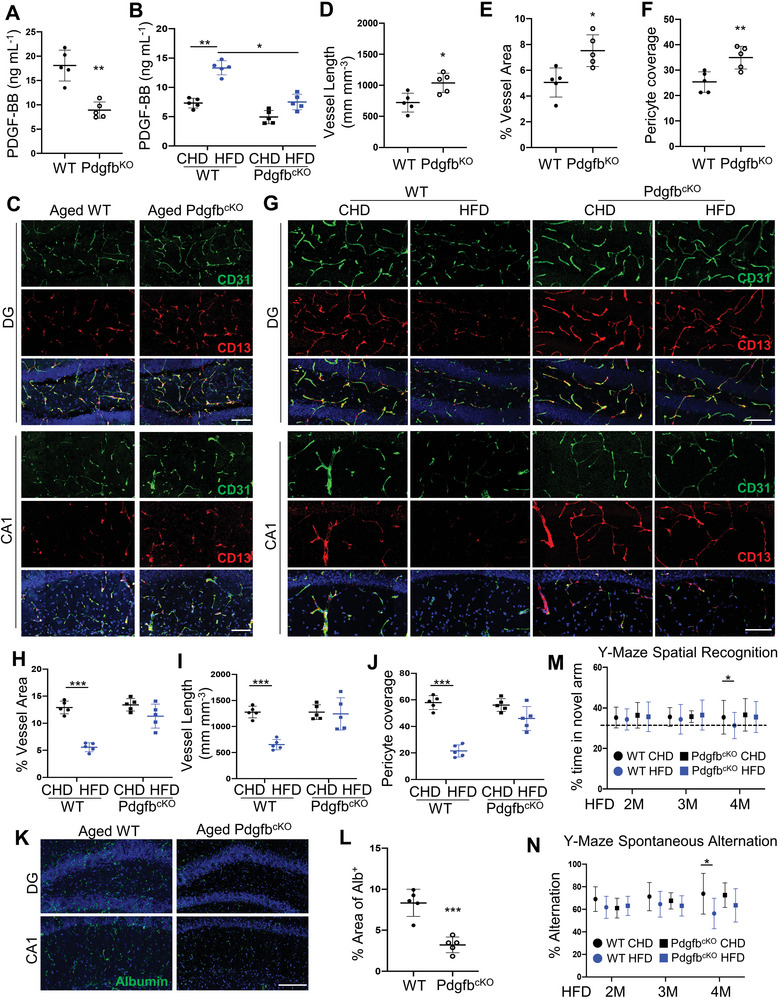
Normalizing the circulating PDGF‐BB ameliorates hippocampal microvascular impairment and cognitive decline. A) ELISA analysis of serum PDGF‐BB concentration in 18‐month‐old Pdgfb^cKO^ mice and WT littermates. B) Pdgfb^cKO^ mice and WT littermates were fed HFD or CHD for 4 months, starting from 3 months of age. ELISA analysis of serum PDGF‐BB concentration. C) Representative confocal images of CD31 (green) and CD13 (red) double‐immunofluorescence staining in DG and CA1 region in 18‐month‐old Pdgfb^cKO^ mice and WT littermates. DAPI stains nuclei blue. Scale bar, 100 µm. D–F) Quantification of the percentage of vessel length (D), vessel area (E), and CD13^+^ pericyte coverage of the capillaries in hippocampus (F), n = 5. G) Representative confocal images of CD31 (green) and CD13 (red) double‐immunofluorescence staining in DG and CA1 region in CHD versus HFD Pdgfb^cKO^ mice. DAPI stains nuclei blue. Scale bar, 100 µm. H–J) Quantification of the percentage of vessel area (H), vessel length (I), and CD13^+^ pericyte coverage of the capillaries in hippocampus (J), n = 5. K) Representative immunofluorescence images of DG and CA1 region of hippocampus from mice of 18‐month‐old Pdgfb^cKO^ mice and WT littermates using antibody against albumin. DAPI stains nuclei as blue. Scale bar, 100 µm. L) Quantification of Albumin^+^ signal covered area using Image J. M,N) Cognitive mouse behaviors in CHD versus HFD Pdgfb^cKO^ mice were assessed. Mice were tested for spatial recognition (M) and spontaneous alternation (N) in Y‐maze. n = 10. *p<0.05, **p<0.01, ***p<0.001 as determined by unpaired two‐tailed Student's *t* test (for 2 group comparison) or One‐way ANOVA (for multiple group comparison).

We also assessed whether normalizing the level of circulating PDGF‐BB using a genetic approach would restore deficits in cerebral vasculature and cognition in aged mice and HFD‐challenged mice. We took advantage of an established conditional *Pdgfb* knockout mice (Pdgfb^cKO^),^[^ ^19^, ^20^, ^24^ ^]^ in which circulating PDGF‐BB level can be reduced by approximately 1/3. While aged WT mice (**Figure** **5**A, Figure S8A, Supporting Information) and HFD‐challenged WT mice (Figure 5B, Figure S8B, Supporting Information) had increased circulating PDGF‐BB concentration, serum and plasma PDGF‐BB concentration was reduced to normal physiological levels in both conditions in Pdgfb^cKO^ mice. Consistent with initial observation, the length and area of the capillaries in hippocampus were reduced in aged WT mice (vs young mice) (Figure 5C–E) and HFD‐challenged mice (vs CHD mice) (Figure 5G–I). However, these deficits were mitigated in the Pdgfb^cKO^ mice under both conditions. Pericyte loss in the hippocampus of aged mice (Figure 5C,F) and HFD mice (Figure 5G,J) was also largely rectified in Pdgfb^cKO^ mice. Furthermore, age‐associated increased permeability as indicated by albumin extravasation was almost abolished in Pdgfb^cKO^ mice (Figure 5K,L). Cognitive measurements showed that WT mice challenged with HFD for 4 months had reduced alternation values and percentage time in the novel arm in the Y‐maze spontaneous alternation and spatial recognition tests, respectively. However, these reductions were not significant in the Pdgfb^cKO^ mice (Figure 5M,N). Therefore, the conditional *Pdgfb* knockout mice with normalized circulating PDGF‐BB levels have much attenuated impairments in hippocampal capillaries and cognitive function during aging or in response to HFD.

### 2.4 Long‐Term High Concentration PDGF‐BB Treatment Leads to PDGFR*β* Shedding in Brain Pericytes

**Figure 6:**
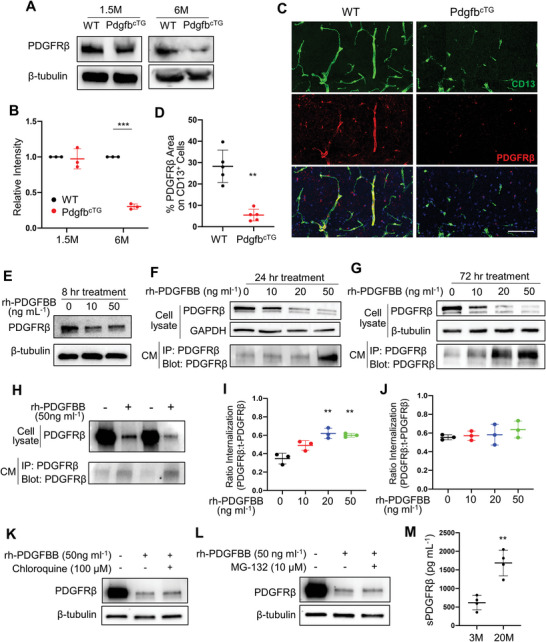
Persistent exposure to high concentrations of PDGF‐BB ligand leads to its receptor shedding in pericytes. A) PDGFR*β* protein expression in hippocampal tissue collected from 1.5‐ and 6‐month‐old Pdgfb^cTG^ mice and WT littermates was assessed by Western blot analysis. B) Quantification of the relative intensity of PDGFR*β* by Image J. n = 3. C) Representative confocal images of CD13 (green) and PDGFR*β* (red) double‐immunofluorescence staining in CA1 region of 6‐month‐old Pdgfb^cTG^ mice and WT littermates. DAPI stains nuclei as blue. Scale bar, 100 µm. D) Quantification of the percentage of PDGFR*β* area on CD13^+^ pericytes in hippocampus. n = 5. E–G) Primary human brain pericytes were treated with different dosages of recombinant human PDGF‐BB (rh‐PDGFBB) for 8 (E), 24 (F), and 72 h (G). PDGFR*β* protein expression was detected by Western blot analysis. H) Primary human brain pericytes were treated with rh‐PDGFBB for 24 h, and culture medium (CM) was collected. PDGFR*β* expression in cell lysate was detected by Western blot analysis (upper panel), and the cleaved sPDGFR*β* in CM was immunoprecipitated by a specific PDGFR*β* antibody followed by Western blot analysis of PDGFR*β* expression (lower panel). I,J) Primary human brain pericytes were treated with rh‐PDGFBB for 24 (I) and 72 h (J), and intracellular ratio was measured by flow cytometry. K,L) Primary human brain pericytes were treated with rh‐PDGFBB together with Chloroquine (K) or MG‐132 (L) for 72 h. PDGFR*β* protein expression was detected by Western blot analysis. n = 3. M) ELISA analysis of CSF sPDGFR*β* concentrations in C57BL/6 mice at 3 and 22 months of age. n = 4. Data are shown as the mean ± SD, **p<0.01, ***p<0.001, unpaired two‐tailed Student's *t* test.

We next attempted to gain mechanistic insights that underline elevated PDGF‐BB ligand‐induced pericyte loss in the hippocampus. As PDGFR*β* is the critical receptor expressed in pericytes to maintain pericytes and BBB homeostasis, we examined the change in PDGFR*β* expression level in the whole brain in young and old WT mice. The result showed that PDGFR*β* expression decreases in hippocampus, increases in thalamus, and has no change in cortex in the 3‐month‐old WT mice versus 21‐month‐old WT mice (Figure S9, Supporting Information), suggesting brain region‐dependent changes in PDGFR*β* expression during aging. Moreover, the distribution patterns of the PDGFR*β* ^+^ cells also appear very different in distinct brain regions. While the majority of the PDGFR*β* ^+^ cells in hippocampus are present along the walls of brain vasculature, most of the PDGFR*β* ^+^ cells in thalamus seem not vascular‐like. We also examined the change in PDGFR*β* expression level in hippocampal tissue from the conditional *Pdgfb* transgenic mice. Western blot analysis showed a reduction in PDGFR*β* protein expression in the brain hippocampus from the Pdgfb^cTG^ mice relative to the WT littermates at 6 months of age (**Figure** **6**A,B). The reduction in PDGFR*β* protein expression in the same brain region was moderate in 1.5‐month‐old Pdgfb^cTG^ mice compared with that in the age‐matched WT mice, indicating a progressive loss of PDGFR*β* in the hippocampus of the conditional transgenic mice. Consistently, double‐immunofluorescence staining revealed a decreased percentage of PDGFR*β*‐expression pericytes out of the total CD13^+^ pericytes in the hippocampus of Pdgfb^cTG^ mice relative to the WT littermates although the absolute number of CD13^+^ pericytes were also reduced in the same region in the transgenic mice (Figure 6C,D). These results suggest that diminished PDGFR*β* expression may be a major contributor to hippocampal pericyte loss.

To test whether PDGF‐BB ligand directly modulates PDGFR*β* expression, we treated primary human brain pericytes with recombinant human PDGF‐BB (rh‐PDGFBB). Although the concentration of PDGF‐BB in CSF is much lower than that in blood,^[^ ^27^ ^]^ pericytes that ensheath capillary brain endothelial cells can directly sense and respond to the changes of the cytokines/inflammatory factors in peripheral blood, especially under the condition of age‐related BBB high permeability. We therefore treated the pericytes with rh‐PDGFBB at a range of serum PDGF‐BB concentrations in aged mice (≈10–50 ng mL^−1^). Our result showed that PDGFR*β* was moderately decreased in pericytes after rh‐PDGFBB treatment at 10 and 50 ng mL^−1^ for 8 h (Figure 6E). Notably, 24‐ and 72‐h rh‐PDGFBB treatment substantially downregulated the level of PDGFR*β* in a dose‐dependent manner (Figure 6F,G, upper two panels), with 72 h‐treatment being more pronounced. The level of PDGFR*β* became barely detectable at 50 ng mL^−1^, suggesting that persistent stimulation with PDGF‐BB at high dosages leads to its receptor loss in pericytes. PDGFR*β* deficiency leads to cell apoptosis of pericytes.^[^ ^28^ ^]^ Indeed, rh‐PDGFBB treatment led to decreased pericyte viability in a dose‐ and time‐dependent manner (Figure S10, Supporting Information).

It has been reported that the extracellular domain of the PDGFR*β* can be proteolytically cleaved from cell membranes of brain pericytes in response to divergent inducers of injury, leading to the release of sPDGRF*β* from the cells.^[^ ^18^, ^29^ ^]^ We also tested whether the reduced level of PDGFR*β* in pericytes is caused by the shedding of this receptor from the cell surface of pericytes. Indeed, whereas Western blot analysis showed diminished level of PDGFR*β* in pericytes in response to 50 ng mL^−1^ rh‐PDGFBB treatment, the levels of cleaved sPDGFR*β* in the culture medium (CM) were increased (Figure 6F–H, bottom panels). Furthermore, dose‐dependent elevation of sPDGFR*β* was observed in CM collected only from 72‐h rhPDGF‐BB‐treated cells (Figure 6G, bottom panel) but not from those with 24‐h treatment (Figure 6F, bottom panel), indicating that a dose‐dependent receptor shedding only occurs in the cells being persistently exposed to PDGF‐BB.

An elegant study by Smyth et al. demonstrated that PDGF‐BB at a physiological concentration (10 ng mL^−1^) triggers the endocytosis of its receptor PDGFR*β*.^[^ ^30^ ^]^ We then tested whether lower doses, shorter period‐treatment of the PDGF‐BB ligand may induce its receptor endocytosis and subsequent protein degradation. As expected, our flow cytometry analysis showed that there is an increase in the ratio of internalized PDGFR*β*/total PDGFR*β* in 24‐h rh‐PDGFBB‐treated cells (Figure 6I and Figure S11, Supporting Information); however, the increase in the receptor internalization was not detected in 72‐h PDGF‐BB‐treated cells (Figure 6J and Figure S12, Supporting Information), suggesting that longer treatment may lead to extracellular domain shedding rather than an internalization of the receptor. We also tested whether antagonizing protein degradation could rescue PDGF‐BB‐induced receptor downregulation. Western blot analysis showed that either a proteasome inhibitor MG132 or a lysosome inhibitor chloroquine failed to raise the level of PDGFR*β* that was reduced by a long‐term (72‐h) rh‐PDGFBB treatment (Figure 6K,L), indicating a protein degradation‐unrelated process. Collectively, our data suggest that although PDGFR*β* undergoes transient endocytosis when the cells are exposed to low concentration PDGF‐BB, persistent exposure to higher concentrations of PDGF‐BB ligand causes the receptor shedding in pericytes. Consistent with these in vitro results, we detected much higher sPDGFR*β* concentration in CSF of aged mice (Figure 6M). However, increased sPDGFR*β* concentration in serum was not detected in aged mice relative to young mice (data not shown). Elevated CSF sPDGFR*β* is a biomarker of BBB dysfunction in Alzheimer's disease patients.^[^ ^18b,c^ ^]^ Our results suggest that increased CSF sPDGFR*β* may also serve as a marker of pericyte loss and BBB impairment in the setting of natural aging.

### 2.5 MMP14 Mediates PDGF‐BB‐Induced PDGFR*β* Shedding from Pericytes

**Figure 7:**
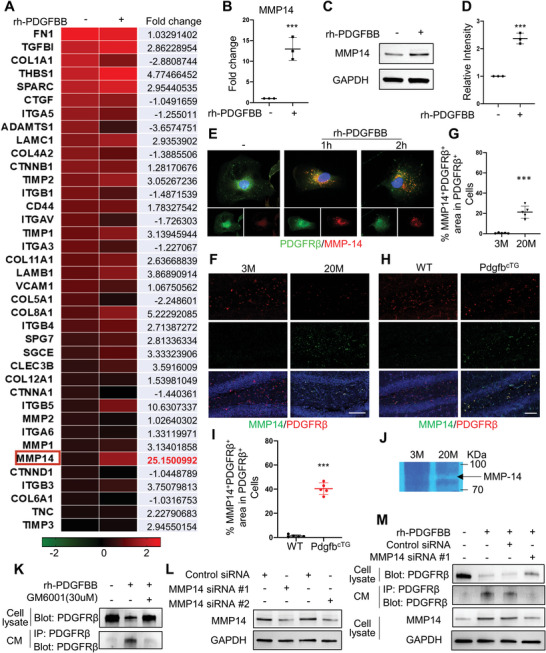
MMP14 mediates PDGF‐BB‐induced PDGFR*β* shedding. A) Gene‐array analysis on rh‐PDGFBB‐treated or control human brain pericytes for 3 days. Heatmaps showed the magnitude of differential expression with fold change (on the right) of each gene. B) Quantitative real‐time PCR analysis of MMP14 mRNA expression in human brain pericyte with or without rh‐PDGFBB treatment for 24 h. n = 3. C) Western blot analysis of MMP14 protein expression in primary human brain pericyte with or without rh‐PDGFBB treatment for 24 h. D) Quantification of the relative intensity of MMP14 using Image J. n = 3. E) Primary human brain pericytes were treated with rh‐PDGFBB for 1 or 2 h. Double‐immunofluorescence staining of the cells was performed using antibodies against MMP14 (red) and PDGFR*β* (green). F,H) Representative confocal images of PDGFR*β* (red) and MMP14 (green) double‐immunofluorescence staining in DG region of 3‐ and 22‐month‐old WT mice (F) and 6‐month‐old Pdgfb^cTG^ mice and WT littermates (H). DAPI stains nuclei as blue. Scale bar, 100 µm. G,I) Quantification of the percentage of MMP14^+^ cells in PDGFR*β* ^+^ pericytes in hippocampus. n = 5. J) Representative images of gel zymography showing the activity of MMP in the hippocampus of 3‐ and 20‐month‐old WT mice. K) Primary human brain pericytes were treated with rh‐PDGFBB in the presence or absence of MMP inhibitor GM6001 for 24 h, and culture medium (CM) was collected. PDGFR*β* expression in cell lysate was detected by Western blot analysis (upper panel), and the cleaved sPDGFR*β* in CM was immunoprecipitated by a specific PDGFR*β* antibody followed by Western blot analysis of PDGFR*β* expression (lower panel). L) Two different MMP14 siRNAs or scrambled control siRNA were individually transfected into human brain pericytes. MMP14 (upper panel) and GAPDH (lower panel) expression levels were detected by Western blot analysis. M) Primary human brain pericytes were transfected by MMP14 siRNA or scrambled control siRNA followed by rh‐PDGFBB treatment for 24 h. PDGFR*β* expression in cell lysate was detected by Western blot analysis (1st Row), and the cleaved sPDGFR*β* in CM was immunoprecipitated by a specific PDGFR*β* antibody followed by Western blot analysis of PDGFR*β* expression (2nd Row). MMP14 (3rd Row) and GAPDH (4th Row) expression levels in cell lysate were also detected by Western blot analysis. Data are shown as the mean ± SD, ***p<0.001, unpaired two‐tailed Student's *t* test.

To determine the key molecule that causes the PDGFR*β* shedding, we conducted an extracellular matrix and adhesion molecule PCR array in PDGF‐BB‐stimulated and ‐non‐stimulated pericytes by profiling 84 genes involved in cell‐cell and cell‐matrix adhesion. Among all genes screened, MMP14 (also named MT1‐MMP) is the most upregulated extracellular molecule (approximately 25‐fold upregulation) in PDGF‐BB‐stimulated pericytes (**Figure** **7**A). The gene array analysis was further validated using both quantitative real‐time PCR and Western blot analysis, in which PDGF‐BB induced upregulation of MMP14 mRNA (Figure 7B) and protein (Figure 7C,D) levels in pericytes. Immunofluorescence staining also showed increased MMP14 accumulation in rh‐PDGFBB‐treated (vs vehicle‐treated) pericytes (Figure 7E). Importantly, rh‐PDGFBB stimulated the co‐localization of MMP14 and PDGFR*β* (Figure 7E), suggesting a physical interaction of these two proteins in pericytes. More importantly, increased MMP14^+^ cells and decreased PDGFR*β* were also observed in hippocampus of aged mice (Figure 7F,G) and Pdgfb^cTG^ mice (Figure 7H,I) compared with young mice and WT mice, respectively. A disintegrin and metalloproteinase 10 (ADAM10) and ADAM17 have been recognized as the main metalloproteinases to cleave PDGFR*β*.^[^ ^31^ ^]^ We also measured ADAM10 and ADAM17 expressions in hippocampus of young and aged mice by Western blot analysis. We detected increased MMP14 and MMP1 expression but unchanged ADAM10 and ADAM17 expression in hippocampus of aged mice as compared with those in young mice (Figure S13, Supporting Information), indicating that these two metalloproteinases may not involve in PDGF‐BB elevation‐induced receptor shedding. Furthermore, gel zymography showed a remarkable increase in MMP14 activity in hippocampus from aged mice (Figure 7J). To test whether inhibiting MMP14 enzymatic activity could antagonize PDGFR*β* shedding from cell membrane of pericytes, we treated the pericytes with rh‐PDGFBB in the presence and absence of an MMP inhibitor GM6001. As expected, GM6001 successfully rescued PDGFR*β* loss in pericytes and elevation of the sPDGFR*β* in the CM induced by PDGF‐BB treatment (Figure 7K). To further confirm the role of MMP14 in mediating PDGFR*β* shedding, MMP14 was knocked down in pericytes using siRNA (Figure 7L). As a result, rh‐PDGFBB‐induced PDGFR*β* loss in pericytes and elevation of the sPDGFR*β* in the CM were largely alleviated by specific MMP14, but not control, siRNA (Figure 7M).

**Figure 8:**
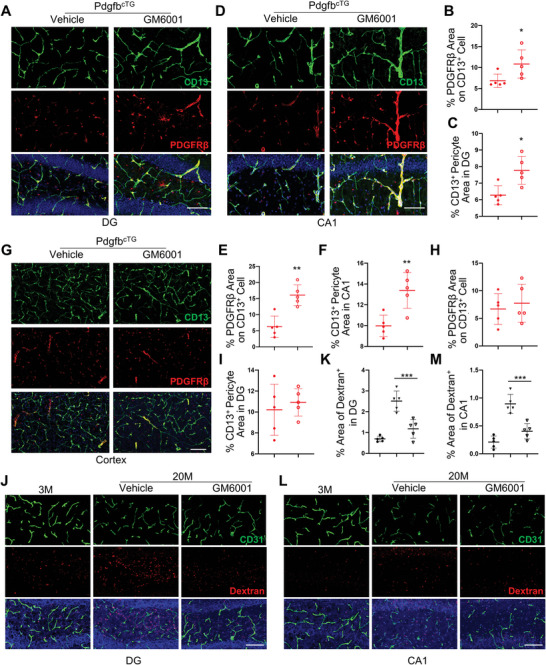
GM6001 rescue MMP14‐induced PDGFR*β* shedding and aged‐associated BBB leakage. A–I) Pdgfb^cTG^ mice were treated with GM6001 or vehicle every other day by i.p. injection for 2 weeks. Representative confocal images of PDGFR*β* (red) and CD13 (green) double‐immunofluorescence staining in DG (A), CA1 (D), and cortex (G). DAPI stains nuclei as blue. Scale bar, 100 µm. Quantification of the percentage of PDGFR*β* expression area on CD13^+^ cells (B, E, H) and CD13^+^ pericyte area (C, F, I) in the three regions. J–M) 18‐month‐old mice were treated with GM6001 or vehicle every other day by i.p. injection for 4 weeks. Representative confocal images of CD31 (green) and Dextran (red) double‐immunofluorescence staining in DG (J) and CA1 (L). DAPI stains nuclei as blue. Scale bar, 100 µm. Quantification of the percentage of Dextran^+^ signal area in these two areas (K and M). n = 5. Data are shown as the mean ± SD, *p<0.05, **p<0.01, ***p<0.001, as determined by unpaired two‐tailed Student's *t* test (for two group comparison) or One‐way ANOVA (for multiple group comparison).

Finally, we evaluated whether inhibition of MMP activity can antagonize age‐associated cerebrovascular impairment in vivo. We first examined the effect of short‐term MMP inhibitor GM6001 treatment on pericyte loss in Pdgfb^cTG^ mice. Daily GM6001 treatment for 2 weeks increased PDGFR*β* expression in both DG (**Figure** **8**A,B) and CA1 regions (Figure 8D,E) but not in cortex (Figure 8G,H) in Pdgfb^cTG^ mice, indicating inhibited receptor shedding by GM6001 in hippocampus. The density of CD13^+^ pericytes was also higher in these 2 regions, but not in cortex, in GM6001‐treated mice relative to vehicle‐treated mice (Figure 8A,C,D,F,G,I), suggesting an attenuation of pericyte loss in hippocampus, not in cortex, induced by PDGF‐BB elevation. We then tested the effects of longer‐term MMP inhibition on cerebrovascular changes in aged mice. BBB permeability was assessed by measuring the relative fluorescence of 10 kDa fluorophore in parenchymal following intravenous injection concurrent with in vivo imaging. While BBB permeability of the fluorophore was significantly increased in 20‐month‐old mice relative to 3‐month‐old young mice, the leakage of the fluorophore in both DG (Figure 8J,K) and CA1 region (Figure 8L,M) was attenuated in 20‐month‐old mice treated with GM6001 for 4 weeks. Thus, inhibition of MMP activity successfully rescued age‐associated phenotype of pericyte loss and BBB impairment.

## 3 Discussion

Blood exchange experiments in heterochronic parabionts or heterochronic plasma transfer have revealed that age‐associated changes in blood composition contribute to neurogenesis and cognitive impairment in the elderly.^[^ ^14^, ^15^, ^32^ ^]^ Blood‐born pro‐aging factors, including *β*2‐microglobulin, transforming growth factor‐β1 (TGF‐*β*1), chemokine (C‐C motif) ligand 11 (CCL11), chemokine (C‐C motif) ligand 2 (CCL2), interlukin‐6 (IL‐6), and tumor necrosis factor‐α (TNF‐*α)*,^[^ ^32a,c–e^ ^]^ have been identified as causal factors to accelerate age‐dependent brain changes. Here, we establish the role of bone‐derived PDGF‐BB as a new member of the group of systemic pro‐aging factors mediating the aging phenotype of brain, particularly hippocampal BBB impairment. Moreover, our studies uncover a previously unidentified molecular mechanism by which persistent high concentration of PDGF‐BB causes brain pericyte loss and cerebrovascular pathology. Increased PDGF‐BB ligand induces upregulation of a matrix metalloproteinase MMP14, which cleaves the extracellular domain of receptor PDGFR*β* from cell membrane of the brain pericytes, leading to diminished PDGF‐BB/PDGFR*β* signaling and the resultant BBB disruption (Figure S14, Supporting Information).

We recently found that aged mice relative to young mice had higher serum PDGF‐BB, which drives age‐associated stiffening of large arteries/macro‐vasculature.^[^ ^20^ ^]^ The finding suggests that PDGF‐BB, a potent angiogenesis factor, maybe a key pro‐aging factor accelerating the aging phenotype of the vascular system in general. Supporting this assumption, the present study, using conditional transgenic and knockout mouse models, demonstrates that abnormally high concentration of PDGF‐BB in blood circulation is both sufficient and necessary to accelerate hippocampal pericyte loss, BBB impairment, and cognitive decline. The major source of PDGF‐BB in blood is platelets upon activation.^[^ ^33^ ^]^ Therefore, the PDGF‐BB concentration in serum is much higher than that in plasma due to PDGF‐BB being released from platelets during serum preparation.^[^ ^21^ ^]^ Our data show that the serum PDGF‐BB concentration in old mice is nearly threefold higher than in young mice, and serum PDGF‐BB concentration in HFD‐challenged mice is 4.9‐fold higher than in normal CHD mice. However, plasma PDGF‐BB is 9.3‐fold higher in old mice (vs young mice) and tenfold higher in HFD (vs CHD mice). The much greater increase of PDGF‐BB concentration in plasma than in serum implies that the excessive circulative PDGF‐BB during aging is likely from source(s) other than blood platelets. We recently revealed that TRAP^+^ skeletal preosteoclasts are a main source of elevated serum PDGF‐BB in aging and under metabolic stress, and the excessive PDGF‐BB in blood circulation mediates arterial stiffening.^[^ ^20^ ^]^ In good agreement with this finding, the present study demonstrates that young skeletal preosteoclast‐specific *Pdgfb* transgenic mice, resemblant to the old mice, have markedly elevated serum and plasma PDGF‐BB concentrations. Moreover, aging‐ or HFD‐induced elevation of serum and plasma PDGF‐BB concentrations were normalized by *Pdgfb* deletion in preosteoclasts. Our results further suggest that skeleton‐derived PDGF‐BB not only contributes to age‐associated functional decline in large arteries but may also drive the impairment of cerebral micro‐vasculature. The mechanisms by which preosteoclasts secrete a high amount of PDGF‐BB during aging or under metabolic stress remain to be determined. Our recent study showed that preosteoclasts undergo cellular senescence and acquire a senescence‐associated secretory phenotype (SASP) in HFD‐challenged mice.^[^ ^34^ ^]^ Notably, PDGF‐BB is one of the SASP factors released from preosteoclasts. Further determining if PDGF‐BB is an important SASP factor released from preosteoclasts during aging will be important to understand the mechanisms through which PDGF‐BB is excessively released during aging and under disease conditions. It has been proposed that there is a bone‐to‐brain axis during the development of Alzheimer's disease^[^ ^35^ ^]^ and Parkinson's disease.^[^ ^36^ ^]^ It is of importance to further investigate the changes in PDGF‐BB/PDGFR*β* signaling in these conditions. A very recent human study reported that elevated levels of circulating PDGF‐BB and loss of PDGF*β* are associated with cerebrovascular damage specifically in individuals at genetic risk of Alzheimer's disease.^[^ ^37^ ^]^ It is interesting to test if elevated PDGF‐BB at the conditions of Alzheimer's disease and small vessel disease also induces PDGFR*β* shedding through MMP14 or other matrix metalloproteinases.

PDGF‐BB/PDGFR*β* signaling is essential for pericyte homeostasis and BBB integrity, and deficiency in either PDGF‐BB ligand availability or its receptor PDGFR*β* leads to pericyte loss and BBB breakdown. Our study, for the first time, reveals that abnormally high circulating PDGF‐BB plays a causal role in age‐dependent impairment of hippocampal BBB and cognitive dysfunction. It is known that too little PDGF‐BB with low PDGF‐BB/PDGFR*β* signaling causes pericyte and BBB impairment. Our findings imply that too much PDGF‐BB also leads to cerebrovascular deficits, providing a novel component to the current understanding of the PDGF‐BB/PDGFR*β* signaling regulation in cerebral vasculature. Therefore, a fine balance of PDGF‐BB levels is needed to maintain the homeostasis of the pericyte and BBB, and either too little or too much PDGF‐BB adversely influences cerebral vasculature. Our results also suggest that the fine balance of PDGF‐BB level may also be important for the maintenance of physiological functions of other tissues. Our earlier studies demonstrated that PDGF‐BB exerts paradoxical effects on bone and large arterial systems depending on the concentration of PDGF‐BB.^[^ ^19^, ^20^ ^]^ Normal physiological range of PDGF‐BB is essential for the maintenance of bone homeostasis in young, healthy mice because deletion of *Pdgfb* from preosteoclasts led to reduced bone mass.^[^ ^19^ ^]^ However, during aging or pathological conditions, aberrantly elevated PDGF‐BB exerts adverse effects on bone and arteries. Conditional transgenic mice (Pdgfb^cTG^), which had approximately threefold higher serum PDGF‐BB concentration, exhibited multiple organ deterioration, including arterial stiffening,^[^ ^20^ ^]^ hippocampal pericyte and BBB impairment (Figure 2F,G and Figure 3), retinal capillary and pericyte loss (data are not shown), and bone loss.^[^ ^20^ ^]^ Taken together, our findings imply that while a physiological PDGF‐BB level is required for the maintenance of tissue homeostasis, persistently high concentrations of PDGF‐BB at pathological levels may lead to multiple organ dysfunction.

It appears that the cerebrovascular impairment in our Pdgfb^cTG^ mice is specifically at hippocampus, and significant capillary reduction and pericyte loss were not detected in cortex and any other tested regions (thalamus and hypothalamus). Increasing evidence from mouse and human studies suggests that brain capillary and BBB permeability can be compromised in a brain region‐dependent manner during aging or in pathophysiological conditions. Based on a recently published work in analysis of the magnitude of vessel loss in 15 different brain regions, vessel loss with aging was significant in 7 out of the 15 regions, with some regions such as hippocampus and the cortex regions that close to hippocampus (i.e., the motor, perirhinal/ecto‐rhinal, somatosensory regions) being more pronounced.^[^ ^38^ ^]^ Consistent with the finding, our results showed reduced capillaries and pericyte loss in both hippocampus and primary somatosensory area in aged relative to young mice. Our data also shows that PDGFR*β* expression only decreases in hippocampus in aged mice versus young mice (Figure S9, Supporting Information). The results suggest that increased PDGF‐BB during aging may downregulate its receptor PDGFR*β* specifically in hippocampus but not in other tested brain regions. One possible explanation for the discrepancy in regional vessel changes between aged mice and our Pdgfb^cTG^ mice is that the Pdgfb^cTG^ mice used to test the vessel changes in this study are at relatively younger age (6‐month‐old). Pericyte loss and BBB leaking have started detectable in hippocampus but not in cortex and other brain regions yet. Supporting this hypothesis, MRI analysis in humans revealed that BBB is compromised initially in the hippocampus during aging.^[^ ^16b^ ^]^ It is important in the future to define whether older Pdgfb^cTG^ mice (e.g., 9 months or older) exhibit pericyte loss and BBB impairment in other brain regions in addition to hippocampus. Another possibility is that PDGF‐BB elevation is not the sole contributor to age‐associated pericyte loss and BBB impairment in certain brain regions. This possibility is supported by the fact that pericytes exhibit phenotypic variability^[^ ^39^ ^]^ and that specialized sub‐populations of pericytes may be differentially distributed throughout the brain.^[^ ^40^ ^]^ This phenotypic diversity could lead to local differences in the response of pericyte to the elevation of PDGF‐BB.

Western HFD, different from the ketogenic diet that is low‐carbohydrate and mimics a condition of starvation,^[^ ^41^ ^]^ has high‐carbohydrate and causes obesity.^[^ ^42^ ^]^ Unlike the neuroprotective and angiogenesis effect of the ketogenic diet,^[^ ^43^ ^]^ HFD has been shown to induce aging brain phenotype, such as pericyte loss, BBB impairment, and cognitive decline.^[^ ^44^ ^]^ It remains controversial whether HFD‐treated mice have changed microvascular density/distribution due to different fat compositions of the diets and treatment durations.^[^ ^22^, ^44^, ^45^ ^]^ In this study, we chose long‐term HFD challenged mice as an additional model to verify the findings from the aging model mainly because HFD‐challenged mice, like aged mice, also have markedly upregulated serum/plasma PDGF‐BB concentrations relative to CHD mice. We found that HFD‐challenged mice exhibited similar BBB phenotype (e.g., reduced brain capillaries, reduced pericyte coverage, BBB leaking) as did in aged mice. We are aware that there are many different other systemic changes (e.g., inflammation, metabolism, endocrine…) in between the HFD‐challenged mice and the aged mice. However, the results from our conditional *Pdgfb* knockout mice (Pdgfb^cKO^) clearly show largely alleviated hippocampal vascular impairment in both aged mice (vs young mice) and HFD mice (vs CHD mice). Thus, normalizing serum/plasma PDGF‐BB levels may be a good strategy to attenuate BBB impairment in general.

One question is how blood PDGF‐BB can cross the brain microvascular endothelial barrier, which is known to have low permeability to most macromolecules.^[^ ^4^, ^46^ ^]^ However, increasing studies have demonstrated that brain aging is sensitive to circulatory proteins.^[^ ^15^, ^32^, ^47^ ^]^ A recent elegant study revealed that there is an age‐related shift in protein transcytosis from ligand‐specific RMT to non‐RMT in aged brain.^[^ ^15c^ ^]^ This shift alters the composition of transcytosing plasma proteins and permits neurotoxic proteins to access the aged parenchyma to exacerbate the aged brain phenotype. Therefore, it is possible that during aging or metabolic dysregulation, there is an early increase in protein transcytosis that permits PDGF‐BB to enter across the endothelial barrier and act on the pericytes or other cells in the brain parenchyma. Indeed, we observed upregulated expression of non‐RMT markers caveolin‐1 and ALPL and diminished expression of RMT receptor TFRC in hippocampal capillaries in our conditional *Pdgfb* transgenic mice and aged mice. Therefore, Pdgfb^cTG^ mice with elevated plasma PDGF‐BB have decreased RMT but increased non‐RMT transcytosis, mimicking age‐related shift in protein transport. The results suggest that elevated plasma PDGF‐BB transmigrates across the endothelium into the pericyte layer likely through the increased non‐RMT transcytosis. It remains to be determined whether age‐associated increase in non‐RMT transcytosis of BBB is induced directly by elevated PDGF‐BB or indirectly by other age‐related factors. We previously found that Pdgfb^cTG^ mice also develop osteoarthritis,^[^ ^24^ ^]^ a skeletal disorder that results in increased circulating inflammatory factors. These factors may increase non‐RMT of BBB to enable the transport of PDGF‐BB across brain endothelium and then act on its receptor PDGFR*β* in pericytes. It is important to verify this assumption in the future. In the present study, we do not intend to emphasize the role of elevated plasma PDGF‐BB as a trigger for pericyte loss and BBB breakdown during aging or under HFD challenge. Instead, our data implies that elevated plasma PDGF‐BB is an important systemic pro‐aging factor to promote/accelerate BBB impairment.

The mechanisms by which persistent elevated PDGF‐BB ligand induces pericyte loss is an important question. A recent work by Smyth et al. demonstrated that the physiological concentration of PDGF‐BB (10 ng mL^−1^) treatment causes a rapid loss of PDGFR*β* due to internalization in pericytes.^[^ ^30^ ^]^ This effect of PDGF‐BB is transient because both cell surface and total PDGFR*β* level returned to baseline in 24–72 h. Our data, using Western blot analyses of the pericyte lysates and the CM in combination with flow cytometry, show the effect of PDGF‐BB ligand on its receptor is concentration‐ and duration‐dependent. Transient exposure to low concentration of its ligand, PDGFR*β* undergoes internalization and transient receptor downregulation on cell membrane, which is consistent with the finding from Smyth et al.^[^ ^30^ ^]^ However, persistent exposure to pathological concentrations of PDGF‐BB ligand (20–50 ng mL^−1^) causes shedding of the receptor and permanent loss of PDGFR*β* signaling in pericytes. Previous series in vitro and in vivo studies demonstrated that the extracellular domain of the PDGFR*β* can be cleaved from cell membrane of brain pericytes in response to hypoxia or injurious stimuli,^[^ ^18^, ^31^ ^]^ and the elevated levels of sPDGFR*β* in CSF positively correlates with BBB disruption in patients with mild cognitive impairment.^[^ ^16^, ^18^ ^]^ Intriguingly, we found that persistent PDGF‐BB treatment can induce its own receptor shedding, and increased sPDGFR*β* was detected in the CM collected from PDGF‐BB‐treated pericytes.

We identified that the cleavage of PDGFR*β* in pericytes is by MMP14, which is upregulated in pericytes in response to PDGF‐BB treatment. Moreover, there was markedly reduced PDGFR*β* expression and increased MMP14 expression in hippocampus of both aged (vs young) mice and Pdgfb^cTG^ (vs WT) mice. It appears that MMP‐expressing cells were both PDGFR*β* ^+^ pericytes and non‐pericytes, indicating cells other than pericytes also have upregulated MMP14. An earlier study using RNA‐seq analysis revealed that pericytes treated with PDGF‐BB have increased expression of many inflammatory factors,^[^ ^30^ ^]^ which may generate an inflammatory local environment. Under inflammatory conditions, the endothelial cells may also express a variety of MMPs. Both MMP inhibitor treatment and MMP14 knockdown by siRNA effectively blocked the shedding of PDGFR*β*. Therefore, an increase in systemic PDGF‐BB, by inducing an MMP14‐mediated receptor shedding as a negative feedback mechanism, may initially prevent the hyperactivation of the PDGF‐BB/PDGFR*β* signaling in brain pericytes. However, prolonged persistent inhibition of PDGF‐BB/PDGFR*β* signaling activation results in loss of pericytes attachment and BBB breakdown (Figure S14, Supporting Information). The mechanisms by which PDGF‐BB induces upregulation of MMP14 remain to be determined. It is interesting to determine whether PDGF‐BB induces the interaction of PDGFR*β* and MMP14 in pericytes. It was reported that ADAM10 and ADAM17, members of ADAM family, are involved in PDGFR*β* shedding in fibroblasts.^[^ ^31^ ^]^ Moreover, it has been reported that elevated CSF levels of ADAM17 exist in subjects with mild cognitive impairment and patients with Alzheimer's disease.^[^ ^48^ ^]^ In our gene array experiment, none of the ADAM family members were upregulated by PDGF‐BB treatment in brain pericytes. Western blot analysis also revealed increased MMP14 expression but unchanged ADAM10 and ADAM17 expressions in hippocampus of aged mice as compared with those in young mice, indicating that these two metalloproteinases may not involve in PDGF‐BB elevation‐induced receptor shedding in our model system. The enzymes responsible for PDGFR*β* shedding are likely tissue‐specific and also dependent on particular physiological context. Finally, we demonstrate that systemic administration of MMP inhibitor GM6001 in our Pdgfb^cTG^ and aged mice rescued pericyte loss and BBB impairment. We are aware that GM6001 is a non‐specific MMP inhibitor that may inhibit the effect of not only MMP14 but also other MMPs. Given that MMP1 is also upregulated in response to PDGF‐BB elevation (Figure 7A and Figure S13, Supporting Information), it is possible that matrix metalloproteinases other than MMP14 are also involved in PDGFR*β* shedding in pericytes in response to PDGF‐BB elevation. However, our results show that the elevation of MMP‐14 is much more dramatic than the elevation of MMP‐1 (25‐fold vs threefold) in rh‐PDGFBB‐treated cells relative to vehicle‐treated control cells (Figure 7A). Moreover, rh‐PDGFBB‐induced PDGFR*β* shedding was almost abolished by MMP14 knockdown using siRNA (Figure 7L,M). Therefore, MMP14 is likely a primary matrix metalloproteinase that is involved in this process. It is technically challenging to fully exclude the involvement of other MMPs, such as MMP1, in rh‐PDGFBB‐induced PDGFR*β* shedding in cerebral vascular aging due to the lack of commercially available inhibitors specific to MMP14. It is necessary to further determine the specific roles of MMP14 and MMP1 in mediating the shedding of PDGFR*β* from pericyte and the BBB alterations associated with aging using selective MMP antibodies or conditional MMP14 or MMP1 knockout mice. In addition, further testing of the beneficial effect of MMP inhibitors on the changes in cognitive function during aging is needed to determine its translational potential.

## 4 Experimental Section

### Mouse Lines

The conditional *Pdgfb* knockout (pdgfb^cKO^) and transgenic mice (pdgfb^cTG^) were generated as described previously.^[^ ^20^, ^24^ ^]^ *Pdgfb^f/f^* mouse strain was purchased from The Jackson Laboratory (Bar Harbor, ME). The *Trap‐Cre* mouse strain was kindly provided by Jolene J. Windle (Virginia Commonwealth University, Richmond, Virginia, USA). *Pdgfb^f/f^* mice were crossed with *Trap‐Cre* mice (homozygous mice for *Pdgfb* flox allele were referred to as “WT” in the text) to generate *Trap‐Cre; Pdgfb^f/f^* mice (referred to as “pdgfb^cKO^” in the text). Pdgfb^cTG^ mouse line was generated by ligation of mouse TRACP5 promoter with 2.8‐kb full‐length human PDGFB cDNA and followed by pronuclear injection in C57BL/6 fertilized eggs. The genotype of the mice was clarified by PCR analyses of genomic DNA isolated from mouse tails using the same primers described previously.^[^ ^20^, ^24^ ^]^ Pdgfb‐tdTomato^cTG^ mouse line was generated by ligation of mouse TRACP5 promoter with 2.8‐kb full‐length human PDGFB cDNA and tdTomato cDNA, and followed by pronuclear injection in C57BL/6 fertilized eggs at Cyagen. Primers used for genotyping the transgenic mice were as follows: Forward: CACTCAGCATCTCATAAAGCTCCTC; Reverse: GTTTATAGGCATGCACCGTGAGAC. Unique product lengths of 223 bp were generated. All animals were housed in the institution's animal facility.

### Brain Tissue Preparation and ELISA Analysis of PDGF‐BB Concentration

Mice were anesthetized with isoflurane (N064L021A, Baxter, USA) before they were sacrificed. The hippocampus, cortex, and thalamus were separated under microscope. The tissue was then homogenized and lysed in RIPA lysis buffer (Thermo Fisher, USA) with Halt Proteinase and phosphatase inhibitor cocktail (Thermo Fisher, USA). 200 µg total proteins per sample were used to detect PDGF‐BB concentration using Mouse/Rat PDGF‐BB Quantikine ELISA Kit (Cat. MBB00 R&D Systems, Inc., Minneapolis, MN, USA) according to instructions in the handbook. Concentration was measured at 450 and 570 nm on a plate reader (Cat. A51119600DPC, Thermo Fisher).

### Serum, Plasma, and CSF Collection and ELISA Analysis of PDGF‐BB and sPDGFR*β* Concentration

After anesthetization, approximately 0.8 mL of blood was collected from the right ventricle of the mice. Serum was obtained by placing whole blood in an empty tube and allowing the blood to clot. Plasma was obtained by placing whole blood into a tube containing 0.5 m EDTA. The samples were centrifuged at 3000 rpm, 4 °C for 15 min. Mouse CSF was collected as previously described.^[^ ^49^ ^]^ Briefly, sharpened glass capillary was inserted into cisterna magna and 5–10 µL of mouse CSF can be collected. Serum, plasma, and CSF were collected and stored at −80 °C until further processing. PDGF‐BB concentration in serum, plasma, and CSF samples was measured using the Mouse/Rat PDGF‐BB Quantikine ELISA Kit (Cat. MBB00 R&D Systems, Inc., Minneapolis, MN, USA) and Mouse PDGFR beta ELISA Kit (Cat. EM60RB, Invitrogen) according to the instruction in the handbook. Concentration was measured at 450 and 570 nm on a plate reader (Cat. A51119600DPC, Thermo Fisher).

### Brain Tissue Processing and Immunofluorescence Staining

After the mice were perfused with PBS and formalin, brains were collected and separated sagittally. One hemisphere was used to dissect the cortex and hippocampus, which was rapidly frozen and stored at −80 °C. Another hemisphere was fixed in 10% buffered formalin phosphate (Cat. SF100‐4 fisher scientific) for 24 h at 4 °C and transferred to 30% sucrose in PBS for another 48 h at 4 °C. Brain tissue was embedded in OCT and cryosectioned coronally at 50 µm with a microtome (Cat. Microm HM 525, Thermo Fisher). For immunofluorescence staining, tissue sections were incubated with specific primary antibodies to mouse PDGFR*β* (1:100, ab32570, Abcam), CD13 (1:100, MCA2183, BIO‐RAD), CD31 (1:50, AF3628 R&D), Caveolin‐1 (1:100, 3267S, Cell signaling Technology), ALPL (1:20, AF2909, R&D), MMP14 (1;100, ab51074, Abcam), Lectin (1:200, DL‐1174, Vector), TFRC (1:100, NB100‐64979, Novus) and Albumin (ab106582, 1:100, Abcam) for 24 h at 4 °C followed by corresponding fluorescence‐linked secondary antibodies (Jackson ImmunoResearch Laboratories) for 1 to 4 h while avoiding light. The sections were then mounted with DAPI (8961S, Cell signaling Technology). The sample images were captured by a confocal microscope (Zeiss LSM 780).

### Vessel Length and Area Analysis

Vessel length analysis was conducted as previously described.^[^ ^8b^ ^]^ Briefly, the length of CD31^+^ capillary was measured using the Image J plug‐in “Neuro J” length analysis tool from the cortex area above hippocampus, whole area of the hippocampus, and 3 randomly similar areas in hypothalamus and thalamus. The length was expressed in mm of CD31^+^ vascular profiles per mm^3^ of brain tissue. The vessel area was calculated as a percentage (%) of CD31^+^ vessel surface area per observation field. In each experimental group, five mice were selected for statistical analysis.

### CD13^+^ Pericyte Coverage Analysis

CD13^+^ pericyte coverage analysis was conducted as previously described.^[^ ^50^ ^]^ Briefly, for pericyte coverage, CD13 (red) and CD31 (green) signals from microvessels were selected and subjected to threshold processing. The areas occupied by the fluorescent signals were measured using ImageJ. The pericyte coverage was calculated as a percentage (%) of CD13^+^ pericyte surface area covering CD31^+^ capillary surface area per observation field. In each experimental group, five mice were selected for statistical analysis.

### BBB Permeability Assessment by Evans Blue and Dextran Injection

Evans Blue (EB; 2% in PBS; Sigma‐Aldrich, E2129) dye or Dextran (Texas Red, 10000MW, D1828, Invitrogen) was injected via tail vein. After 2 h, mice were euthanized and perfused with PBS. For dextran leakage, brain was fixed and cryosectioned as above. Dextran fluorescence brain section was mounted with DAPI and observed using confocal microscope.

For EB leakage, after perfusion, brain tissue was harvested, then hippocampus, cortex, and thalamus were dissected and weighed. EB leakage was quantified in the tissue after the brain tissue had been incubated in formamide (48 h, 55 °C). The EB assay result was measured in the supernatant from each sample (absorbance, 610 nm). The results were calculated using a standard curve of EB in formamide and were presented as micrograms per gram of brain tissue.

### MRI Measurements

All MRI experiments were performed on an 11.7T Bruker Biospec system (Bruker, Ettlingen, Germany) equipped with a horizontal bore and actively shielded pulsed field gradients (maximum intensity of 0.74 T m^−1^). MRI experiments and data analyses were performed in a double‐blind manner. A cohort of 5 Pdgfb^cTG^ (6 months) and 5 WT littermates (6 months) was scanned under randomized orders. MRI images were acquired using a 72‐mm quadrature volume resonator as transmitter, and a four‐element (2×2) phased‐array coil as receiver. The B_0_ field over the mouse brain was shimmed with a global shimming scheme (to 2nd order) based on a subject‐specific pre‐acquired field map. Anesthesia was carried out by medical air (21% O_2_, 78% N_2_) with 1.5% vaporized isoflurane for 15 mins as induction, then 1.0% for maintenance until the end of experiments. Respiration rate was monitored during the experiment to ensure the survival of the mouse. In case that a mouse breathed at a rate >150 breaths per minute, the maintenance isoflurane dosage was increased slightly to ≈1.2%. For MRI scanning, the mouse was immobilized with a bite bar and ear pins and then placed on a water‐heated animal bed with temperature control. Brain volume was estimated from a T_2_‐weighted fast‐spin‐echo anatomy MRI scan under the following parameters: TR/TE = 4000/26 ms, FOV = 15×15 mm^2^, matrix size = 128×128, slice thickness = 0.5 mm, echo spacing = 5 ms, 35 axial slices, and scan duration = 1.1 min. The WEPCAST MRI was performed under the following parameters: TR/TE = 3000/13.1 ms, FOV = 15×15 mm^2^, matrix size = 96×96, slice thickness = 1.0 mm, band width = 300 kHz, labeling duration = 1800 ms, post‐labeling delay = 300 ms, and scan duration = 5 min with the two‐segment spin‐echo echo‐planar‐imaging (EPI) acquisition. For estimating the inversion efficiency (denoted as *α*) of the labeling module in WEPCAST MRI, phase‐contrast MRI following the previous report^[^ ^51^ ^]^ was performed for normalization.^[^ ^52^ ^]^ Data processing for anatomy MRI and phase contrast MRI was performed as reported.^[^ ^51^ ^]^ WEPCAST MRI data were processed as follows: difference images (M_Diff_) were obtained with pair‐wise subtraction between control and labeled images; M_0_ image was estimated from control image by accounting for the longitudinal magnetization recovery; water retaining fraction (WRF) was calculated based on the WEPCAST signal model.^[^ ^16a^ ^]^

### Behavior Testing

Behavior testing was conducted by the Johns Hopkins Behavior Core.

### Open Field Test

Mice were placed in an open‐field chamber (40×40×38 cm) for 30 min under low lighting conditions. The anxiety was assessed as the total center beam breaks and total peripheral beam breaks using Photobeam Activity System (San Diego Instruments Inc., San Diego, CA) computer software.

### Y‐MAZE Spontaneous Alternation

The spontaneous alternation test was performed in a symmetrical yellow Y‐Maze with three arms (60 cm long × 10 cm wide × 15 cm high) at 120° angles, named A, B, and C. The mice were placed in the distal end of arm A and allowed to explore the maze for 5 min. A video camera mounted above the maze was used to record the movements of the mice for analysis. The video was recorded by a ceiling‐mounted camera and the percentage of alternations (entry into an arm that differs from the previous two entries) was calculated by a ceiling‐mounted Any‐maze tracking software (Stoelting Co., Wood Dale, IL, USA). Data were expressed as a percentage of alternation during the total 5 min.

### Y‐MAZE Spatial Recognition

This test consisted of two trials separated to assess spatial recognition memory and was performed in a symmetrical yellow Y‐Maze with three arms (60 cm long × 10 cm wide × 15 cm high) at 120° angles, named arm 1, arm 2 and novel arm. The first trial (training) had a 5‐min duration and allowed the mouse to explore only arm 1 and arm 2 of the maze, with the novel arm being blocked. After 30 min, the second trial (retention) was performed, during which all three arms were accessible and the percentage of time in novel arm was analyzed by comparing behavior in all three arms. For the second trial, the mouse was placed back in the same starting point of maze, with free access to all three arms for 5 min. The video was recorded by a ceiling‐mounted camera and the percentage of alternations (entry into an arm that differs from the previous two entries) was calculated by a ceiling‐mounted Any‐maze tracking software (Stoelting Co., Wood Dale, IL, USA). Data were also expressed as percentage time spent in novel arms during the total 5 min.

### Novel Object Test

Mice were tested in 20 × 20 cm boxes. Each mouse was allowed to familiarize with the box for 10 min on Day 1. On Day 2, each mouse was placed in the box with two identical objects (cup or whistle) and allowed to explore for 10 min. After 10 min, the mouse was removed and placed back in the home cage. 30 min later, one of the objects was replaced with a novel object. The mouse was placed back in the box and allowed to explore for 5 min. Time spent investigating the objects was measured using Cleversys Topscan automatic tracking software (Clever Sys Inc., Reston, VA, USA).

### Zymography

Zymography was performed according to the previous study with modifications.^[^ ^53^ ^]^ Briefly, protein extracts from murine hippocampus tissue were isolated. 100 µg of total protein was loaded per sample and run on zymography gel (2% gelatin gel). Gel was developed for 40 h and stained in 0.05% Coomassie Blue solution. The images of the gel were captured on scanner.

### Primary Human Pericyte Culture and Treatments

Primary human brain pericyte was purchased from Cell Systems (Seattle, WA). Fourth to seventh passages of the cells were used for all the experiments. Cells were seeded at 4 × 10^4^ cm^−2^ in Complete Classic Medium (Cell systems) with 10% serum and Culture Boost (Cell Systems). In some experiments, cells were incubated with human recombinant PDGF‐BB (rh‐PDGFBB) (R&D System) or MMP inhibitor GM6001 (Cat. 3537171, Millipore Sigma), MG132 (#2194, Cell signaling Technology), and Choloroquine (#14774, Cell signaling Technology), respectively. For siRNA knockdown experiments, cells were transfected with scrambled control siRNA or specific MMP14 siRNA by using Lipofectamine RNAiMAX (Thermo Fisher) according to manufacturer's instructions at 50% confluency. After 36 h transfection, cells were treated with rh‐PDGFBB in different growth mediums containing 5% or 10% serum. Culture medium (CM) and cell lysate were prepared after 24 h incubation, and immunoprecipitation and Western blot analysis were performed as described below.

### Cell Proliferation Assay

Primary human brain pericytes were seeded in 96‐well plate at 70% confluency in culture medium and incubated overnight at 37 °C. Different concentrations of rh‐PDGFBB were added and cultured for 24, 48, or 72 h. Cell proliferation was assayed using MTT assay kit (Roche, 11465007001) according to the manufacturer's instructions. MTT assays were performed in quadruplicates each time and repeated three times.

### Preparation of CM and Immunoprecipitation

Primary human brain pericytes were incubated with a mixture of 50% serum‐free medium and 50% Complete Classic Medium with or without rh‐PDGFBB. After 24 h, the medium was collected and centrifuged at 1500 rpm for 5 min at room temperature. The supernatant was collected and stored at −80 °C as CM.

CM prepared as described above was subjected to immunoprecipitation using a Pierce Co‐Immunoprecipitation Kit (Cat. 26149, Thermo Fisher, USA). For antibody‐bead coupling, 50 µL AminoLink Plus Coupling Resin and 2 µg of PDGFR*β* antibody (goat anti‐human, Cat. AF385, R&D, USA) were mixed with rotating for 30 min at room temperature. Culture medium were added to the antibody/bead mixture and incubated for 1 h with rotation at room temperature. Target antigen was eluted in 50µl denaturing conditions and quantitative Western immunoblot was performed as described below.

### Flow Cytometry Analysis

Flow cytometric analysis from primary human brain pericyte was performed as previously described^[^ ^30^ ^]^ with modifications. Briefly, the primary human brain pericyte was treated with PDGF‐BB (10, 20, and 50 ng mL^−1^) for 1 or 3 days. Then cell was washed with PBS for 3 times and dislocated using StemPro Accutase (5 min, 37 °C, Gibco).

For cell‐surface labeling of PDGFR*β*, cells were centrifuged (1000 rpm, 5 min) and resuspended in cold flow cytometry buffer (0.5% FBS in PBS) as a wash. After washing, equal numbers of cells were incubated for 30 min at 4 °C with PDGFR*β* antibody (1:100, AF385, R&D). After washing 3 times, cells were incubated with FITC‐conjugated second antibody (Jackson ImmunoResearch Laboratories) against PDGFR*β* for 30 min. After washing, cells were analyzed using a BD LSRII flow cytometer.

To measure total PDGFR*β* expression, cells were harvested using Accutase as above. Then fixed by 4% PFA for 15 min and washed twice in 0.1% PBST to permeabilize. After washing with PBST, cells were incubated overnight at 4 °C on shaker with PDGFR*β* antibody (1:100, AF385, R&D) and followed by incubating with FITC‐conjugated second antibody (Jackson ImmunoResearch Laboratories) against PDGFR*β* for 1 h. After washing, cells were analyzed using a BD LSRII flow cytometer.

### Western Blot Analysis

Western blot analysis was conducted as previously described.^[^ ^54^ ^]^ Briefly, the hippocampal, cortex, and thalamus tissue was isolated from mice and homogenized. Tissues and cells were lysed in RIPA lysis buffer (Thermo Fisher, USA) with Halt Proteinase and phosphatase inhibitor cocktail (Thermo Fisher, USA). 30 µg protein was separated by SDS–PAGE and electro‐transferred onto a nitrocellulose membrane. After blocking, the membrane was incubated with primary antibodies at 4 °C overnight and then with secondary antibodies at RT for 1 h. Proteins were visualized with Supersignal West Pico Femto Maxium Sensitivity Substrate (Cat. 34094, Thermo Fisher, USA) and detected with Chemiluminescence Apparatus (Cat. CL‐1500, Thermo Fisher, USA). Statistical analysis was performed using iBright analysis software V 4.0.0. The following primary antibodies were used: PDGFR*β* (1:5000, ab32570, Abcam) (used on cell lysate experiments), Human PDGFR*β* Antibody (1:2000, AF385, R&D; 1:1000, 10514‐RP02‐50, Sino Biological) (used on CMs experiment), *β*‐Tubulin (1:1000, #2128, Cell signaling Technology), GAPDH (1:1000, #5174, Cell signaling Technology), MMP14 (1:5000, ab51074, Abcam), MMP1 (1:1000, PA5‐27210, Invitrogen), ADAM10 (1:5000, ab124695, Abcam), and ADAM17 (1:500, PA5‐27395, Invitrogen).

### Immunocytochemistry

Primary human brain pericytes were treated with rh‐PDGFBB for 1 or 2 h. After washing with PBS for 3 times, cells were incubated with specific primary antibodies to PDGFR*β* (1:100, ab32570, Abcam) and MMP14 (1:100, ab51074, Abcam) for 24 h at 4 °C followed by corresponding fluorescence‐linked secondary antibodies (Jackson ImmunoResearch Laboratories) for 1 h while avoiding light. The sample images were captured by a confocal microscope (Zeiss LSM 780).

### RNA Purification, and Quantitative Real‐Time PCR, and Gene Array Analysis

Total RNA for qRT‐PCR was purified from the cultured or sorting cells using TRIzol (Invitrogen, 15596026) followed by RNeasy Mini Kit (QIAGEN, 74014) according to the manufacturer's protocol. Complementary DNA (cDNA) was prepared with random primers using the SuperScript First‐Strand Synthesis System (Invitrogen) and analyzed with SYBR Green Master Mix (QIAGEN) in the thermal cycler with 2 sets of primers specific for each targeted gene. Target‐gene expression was normalized to glyceraldehyde 3‐phosphate dehydrogenase (GAPDH) messenger RNA, and relative gene expression was assessed using the 2^−ΔΔCT^ method. Primers used for qRT‐PCR were as follows: MMP14: Forward: 5’‐GGCTACAGCAATATGGCTACC‐3’, Reverse: 5’‐ GATGGCCGCTGAGAGTGAC‐3’. GAPDH: Forward: 5’‐ GGAGCGAGATCCCTCCAAAAT‐3’, Reverse: 5’‐ GGCTGTTGTCATACTTCTCATGG‐3’.

For gene array analysis, RNA was purified and reversed using Qiagen RNeasy Plus Kit (Cat. 74034, Qiagen) and PrimeScript RT Master Mix (Cat. RR036B, Takara, Japan) according to manufacturer's instructions. Then, cDNA was subjected to gene array analysis using Human Extracellular Matrix & Adhesion Molecules array (Cat. 4414133, Thermo Fisher, USA) according to manufacturer's instructions. Z‐score standardizing was performed using Excel with mathematical formula:

(1) $$z=\left(X-\mu \right)/\sigma$$

where:

X is a single raw data value
µ is the mean of the dataset
*σ* is the standard deviation of the dataset

The data were displayed as heatmaps using GraphPad Prism 8 software, and the number on the left side of the heatmap was a fold change of rh‐PDGF‐BB group compared with control group.

### Statistics

Data were analyzed using GraphPad Prism 8 statistical software (GraphPad Software Inc., La Jolla, CA). An unpaired two‐tailed Student's *t* test was used to evaluate the statistical difference between 2 groups. In cases of multiple groups, differences were analyzed with one‐way ANOVA with Turkey's multiple comparisons. For analysis using primary human microvascular pericyte, experiments were repeated independently at least 3 times. For the scatter plot, Pearson's correlation tests were used to test for statistical significance. Values of *P<0.05, **P<0.01, and ***P<0.001 were considered statistically significant.

## Conflict of Interest

The authors declare no conflict of interest.

## Author Contributions

G.L. and J.W. contributed equally to this work. G.L. and M.W. designed the experiments, analyzed results, and wrote the manuscript; G.L. and J.W. carried out most of the experiments; Z.W., C‐L.F., K.S., C.Q., and C.Q. helped with some experiments; M.W. supervised the experiments; X.C., P.W., H.L., P.G., and T.L. proofread the manuscript.

## Supporting information

Supporting Information

## Data Availability

The data that support the findings of this study are available from the corresponding author upon reasonable request.
