# Supplementary material for: Elevated PDGF‐BB from Bone Impairs Hippocampal Vasculature by Inducing PDGFRβ Shedding from Pericytes
Source: Adv Sci (Weinh). 2023 Apr 27;10(20):2206938. doi: 10.1002/advs.202206938 (PMC10369301; doi:10.1002/advs.202206938)
Supplement: Supplementary file 1 — Supporting Information [file ADVS-10-2206938-s001.pdf]

ADVANCED SCIENCE
Open Access

Supporting Information

for *Adv. Sci.*, DOI 10.1002/advs.202206938

Elevated PDGF-BB from Bone Impairs Hippocampal Vasculature by Inducing PDGFRβ Shedding from Pericytes

*Guanqiao Liu, Jiekang Wang, Zhiliang Wei, Ching-Lien Fang, Ke Shen, Cheng Qian, Cheng Qi, Tong Li, Peisong Gao, Philip C. Wong, Hanzhang Lu, Xu Cao and Mei Wan**

Supporting information

Elevated PDGF-BB From Bone Impairs Hippocampal Vasculature by Inducing PDGFRβ Shedding from Pericytes

*Guanqiao Liu, Jiekang Wang, Zhiliang Wei, Ching-Lien Fang, Ke Shen, Cheng Qian, Cheng Qi, Tong Li, Philip C. Wong, Hanzhang Lu, Xu Cao, and Mei Wan**

Figure S1

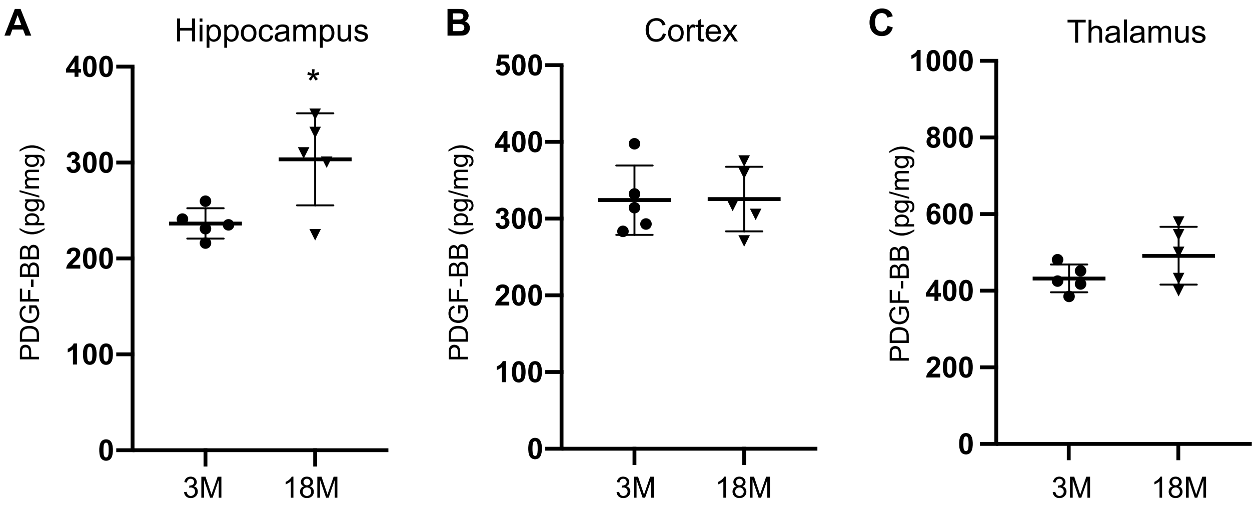

Figure S1. PDGF-BB is only elevated in hippocampus during aging. ELISA analysis of PDGF-BB concentrations in protein extracts from hippocampus (**A**), cortex (**B**) and thalamus (**C**) of C57BL/6 mice at 3 and 18 months of age. n=5. Data are shown as the mean ± SD, *p<0.05, as determined by unpaired two-tailed Student's *t* test.

Figure S2

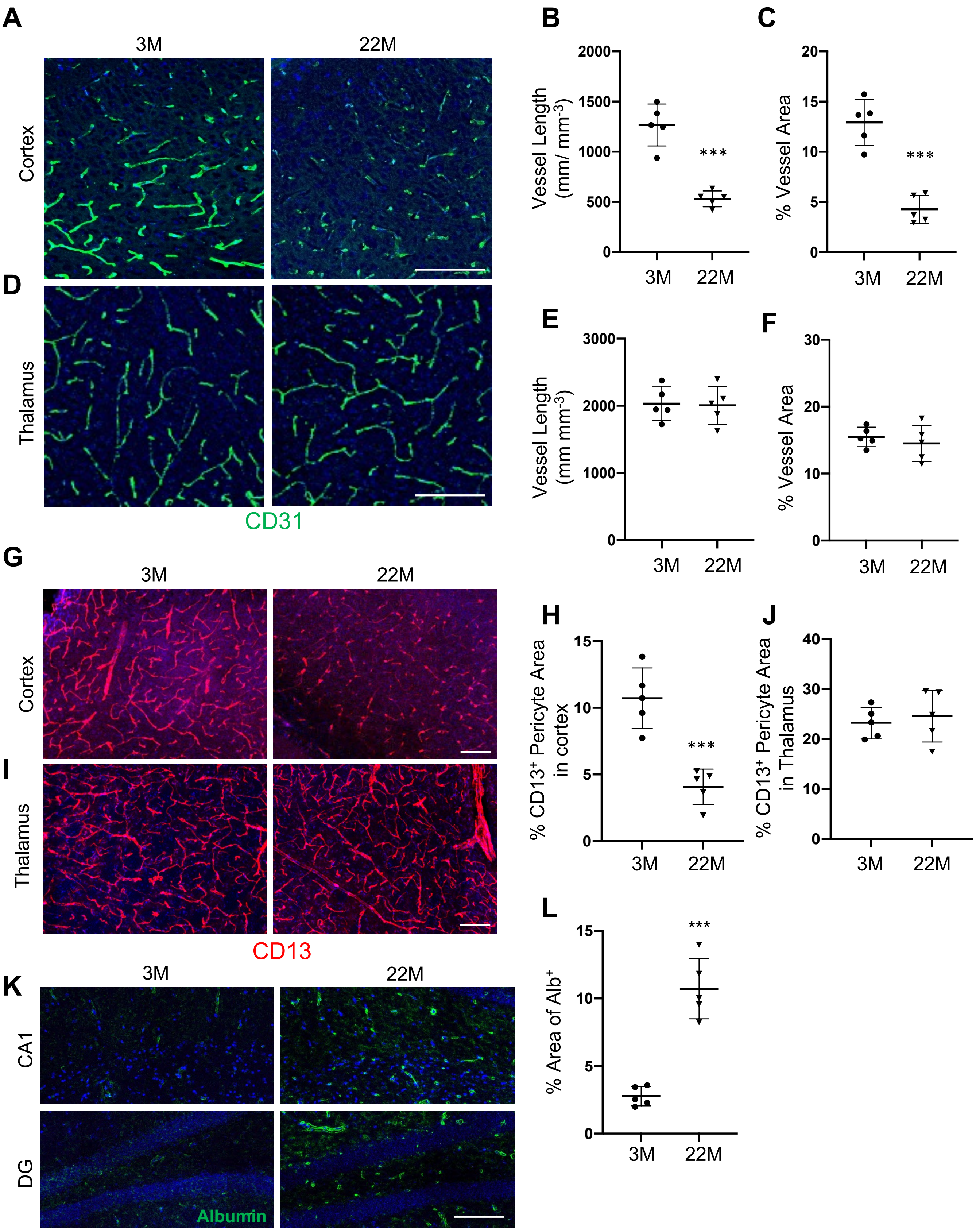
A
3M
22M
Cortex
B
2000
1500
1000
500
0
Vessel Length (mm/ mm-3)
***
3M
22M
C
20
15
10
5
0
% Vessel Area
***
3M
22M
D
Thalamus
CD31
E
3000
2000
1000
0
Vessel Length (mm mm-3)
3M
22M
F
30
20
10
0
% Vessel Area
3M
22M
G
3M
22M
Cortex
I
Thalamus
CD13
H
15
10
5
0
% CD13+ Pericyte Area in cortex
***
3M
22M
J
40
30
20
10
0
% CD13+ Pericyte Area in Thalamus
3M
22M
K
3M
22M
CA1
DG
Albumin
L
15
10
5
0
% Area of Alb+
***
3M
22M

Figure S2. Microvascular impairment and pericyte loss occurred in cortex during aging. Representative confocal images of CD31 (green) and CD13 (red) staining in cortex (**A** and **G**) and thalamus (**D** and **I**) from 3- and 22-months old WT mice. DAPI stains nuclei blue. Scale bar, 100 μm. Quantification of percentage of vessel length (**B**) and vessel area (**C**) in cortex. Quantification of percentage of vessel length (**E**) and vessel area (**F**) in thalamus. Quantification of $CD13^{+}$ signal covered area in cortex **(H)** and thalamus **(J)** using Image J. **(K)** Representative immunofluorescence images of DG and CA1 region of hippocampus from mice of 3 and 22 months of age using antibody against albumin. DAPI stains nuclei as blue. Scale bar, 100 μm. **(L)** Quantification of $Albumin^{+}$ signal covered area using Image J. n=5. Data are shown as the mean ± SD, ***$p<0.001$, as determined by unpaired two-tailed Student's *t* test.

Figure S3

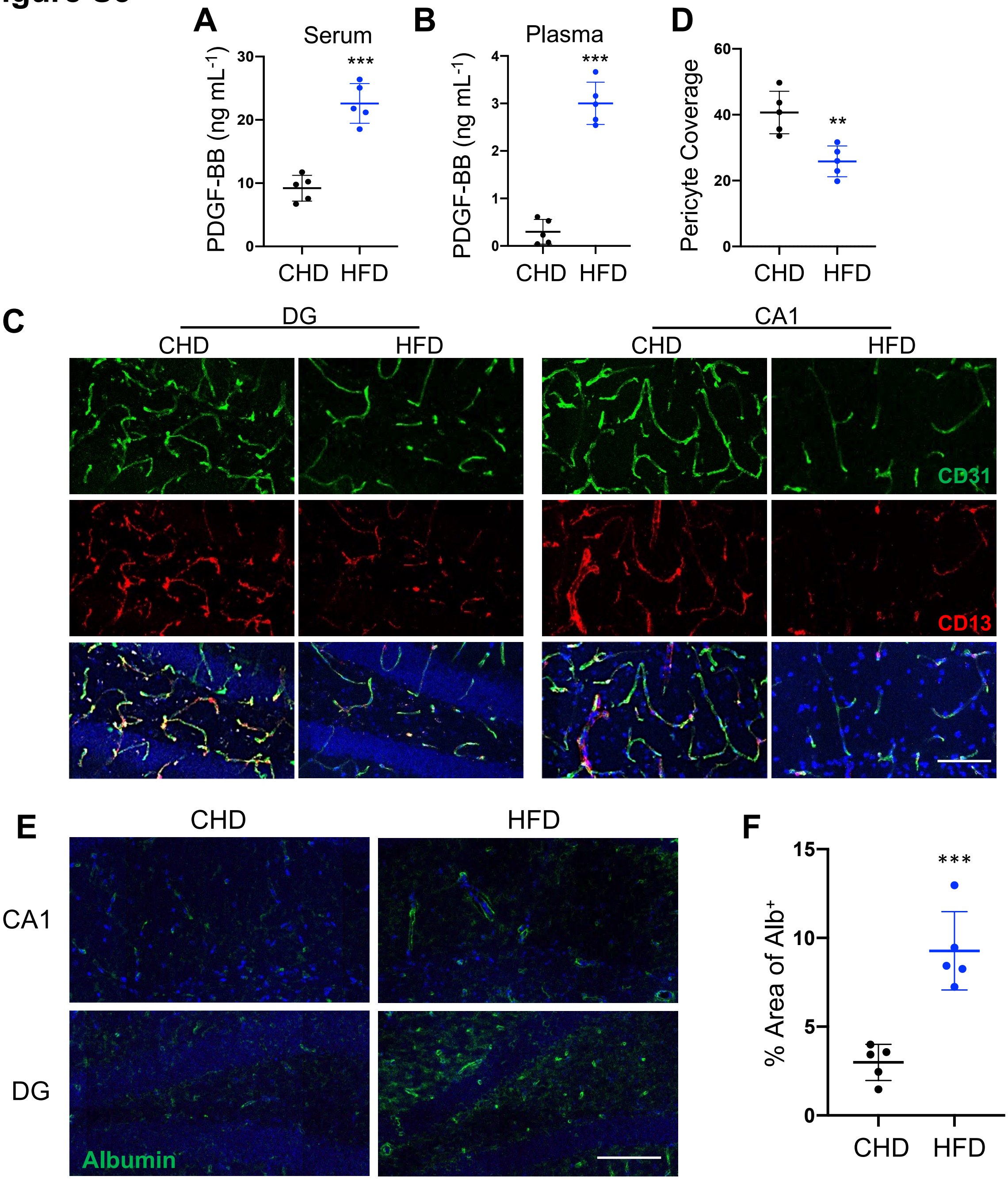

Figure S3. Mice fed with HFD recapitulate aged BBB phenotype ELISA analysis of serum **(A)** and plasma (**B**) PDGF-BB concentration in 3-month-old C57BL/6 mice fed a Western HFD or normal CHD for 4 months. Representative confocal images of CD31 (green) and CD13 (red) staining in DG and CA1 region of 3-month-old mice fed HFD and normal CHD for 4 months (**C**). DAPI stains nuclei blue. Scale bar, 100 μm. Quantification of $CD13^+$ pericyte coverage of the capillaries in hippocampus from mice fed with CHD and HFD (**D**). Representative immunofluorescence images of DG and CA1 region of hippocampus from mice fed CHD and HFD using antibody against albumin (**E**). DAPI stains nuclei as blue. Scale bar, 100 μm. Quantification of $Albumin^+$ signal covered area using Image J (**F**). n=5. Data are shown as the mean ± SD, **p<0.01, ***p<0.001, as determined by unpaired two-tailed Student's *t* test.

Figure S4

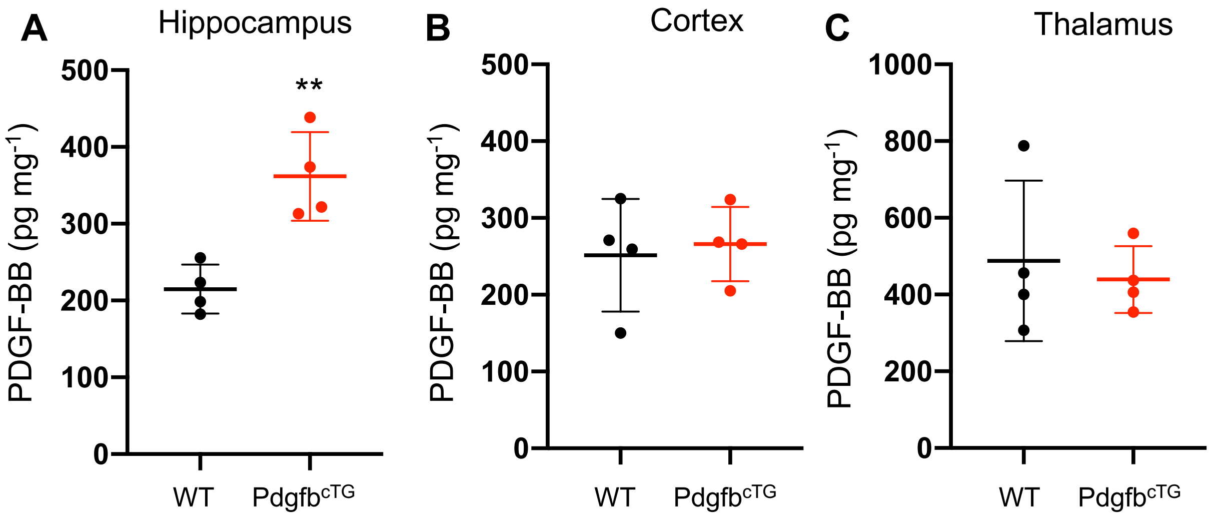

Figure S4. PDGF-BB is only elevated in hippocampus in conditional *Pdgfb* transgenic mice. ELISA analysis of PDGF-BB concentrations in protein extracts from hippocampus (**A**), cortex (**B**) and thalamus (**C**) of Pdgfb^{cTG} mice and WT littermates. n=4. Data own as the mean ± SD, **p<0.01, as determined by unpaired two-tailed Student's *t* test.

Figure S5

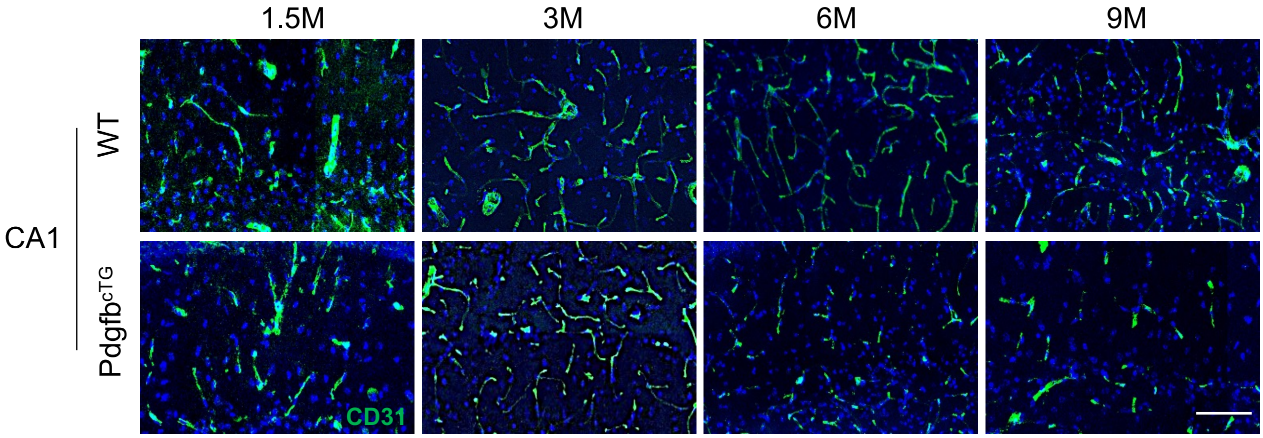

Figure S5. Conditional *Pdgfb* transgenic mice have decreased integrity and density of the capillaries in CA1 region. Representative confocal images of CD31 (green) immunofluorescence staining in CA1 region of Pdgfb^{cTG} mice and WT littermates at 1.5, 3, 6, 9 months of age. DAPI stains nuclei as blue. Scale bar, 100 µm.

Figure S6

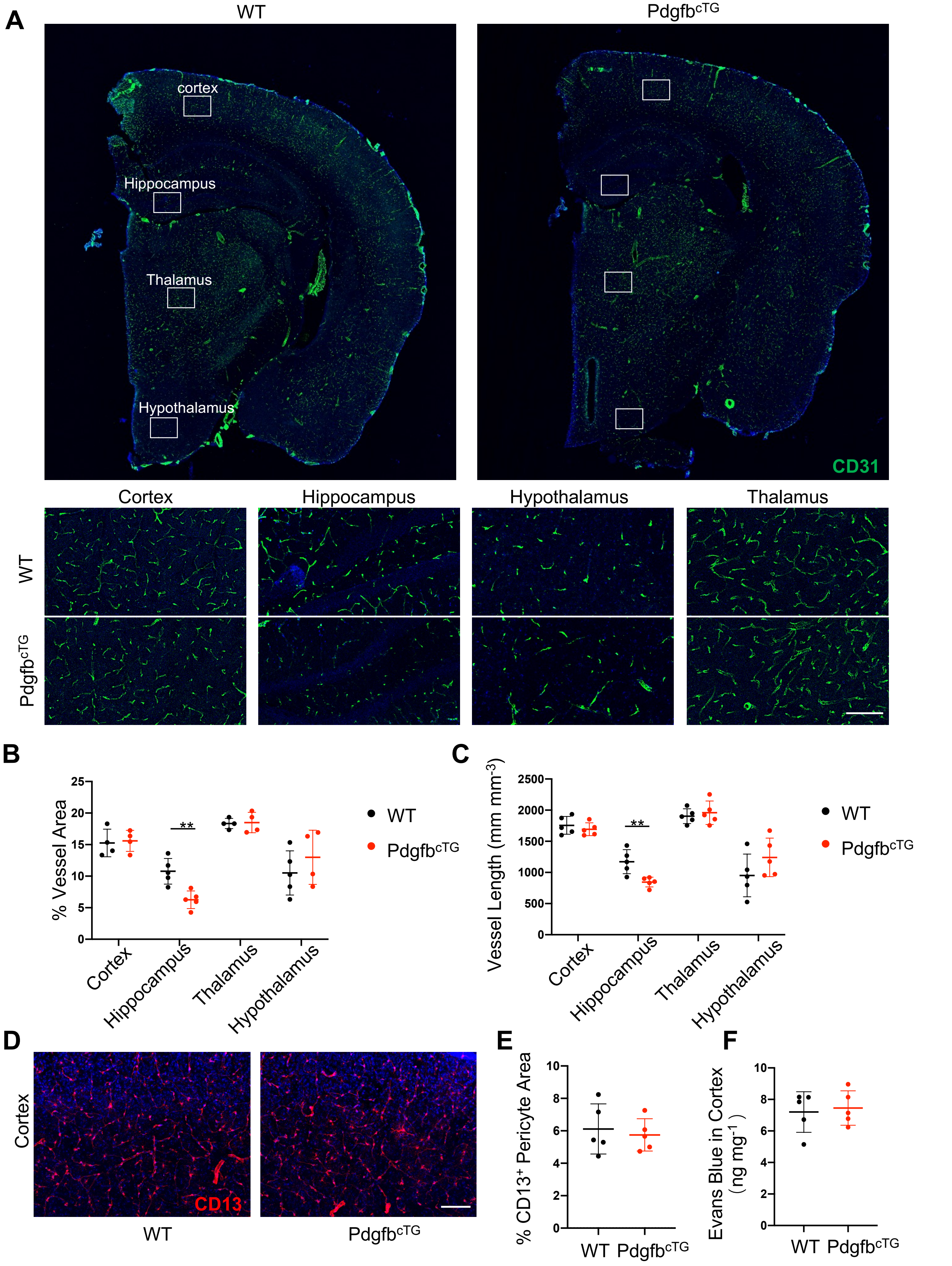
A
WT
PdgfbcTG
cortex
Hippocampus
Thalamus
Hypothalamus
CD31
Cortex
Hippocampus
Hypothalamus
Thalamus
WT
PdgfbcTG
B
% Vessel Area
25
20
15
10
5
0
**
Cortex
Hippocampus
Thalamus
Hypothalamus
WT
PdgfbcTG
C
Vessel Length (mm mm-3)
2500
2000
1500
1000
500
0
**
Cortex
Hippocampus
Thalamus
Hypothalamus
WT
PdgfbcTG
D
Cortex
CD13
WT
PdgfbcTG
E
% CD13+ Pericyte Area
10
8
6
4
2
0
WT
PdgfbcTG
F
Evans Blue in Cortex (ng mg-1)
10
8
6
4
2
0
WT
PdgfbcTG

Figure S6. Hippocampus-specific capillary impairment in Pdgfb^{cTG} mice. Representative CD31 immunofluorescence of whole brain images (upper) and enlarged images of different regions (lower) from 6 months Pdgfb^{cTG} mice and WT littermates (**A**). DAPI stains nuclei blue. Scale bar, 100 μm. Quantification of percentage of vessel area (**B**) and vessel length (**C**) in hippocampus, cortex, thalamus and hypothalamus area. n=4-5. Representative immunofluorescence images of CD13^{+} pericyte area in cortex from Pdgfb^{cTG} mice and their WT littermates (**D**). DAPI stains nuclei as blue. Scale bar, 100 μm. Quantification of CD13^{+} signal covered area using Image J (**E**). n=5. In vivo Evans blue permeability assay in cortex in 6-month-old Pdgfb^{cTG} mice and WT littermates **(F)**. n=5. Data are shown as the mean ± SD, $^{**}p<0.01$, as determined by unpaired two-tailed Student's *t* test.

Figure S7

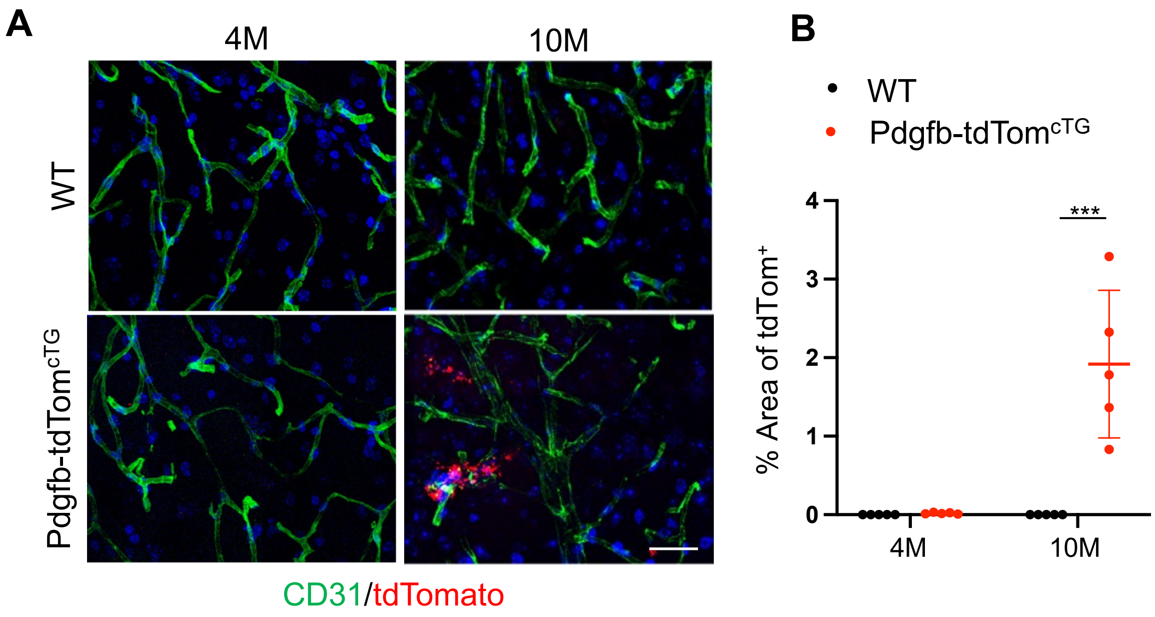

Figure S7. Persistently increased PDGF-BB derived from preosteoclasts can penetrate the endothelium to brain. tdTomato-PDGF-BB produced from bone TRAP+ cells (in red) was detected in brain parenchyma only in 10-month-old Pdgfb-tdTom^{cTG} mice but not in 4-month-old Pdgfb-tdTom^{cTG} mice(**A**). Quantification of tdTom^{+} signal covered area using Image J (**B**). n=5. DAPI stains nuclei as blue. Scale bar, 50μm. Data own as the mean ± SD, **p<0.01, as determined by unpaired two-tailed Student's *t* test.

Figure S8

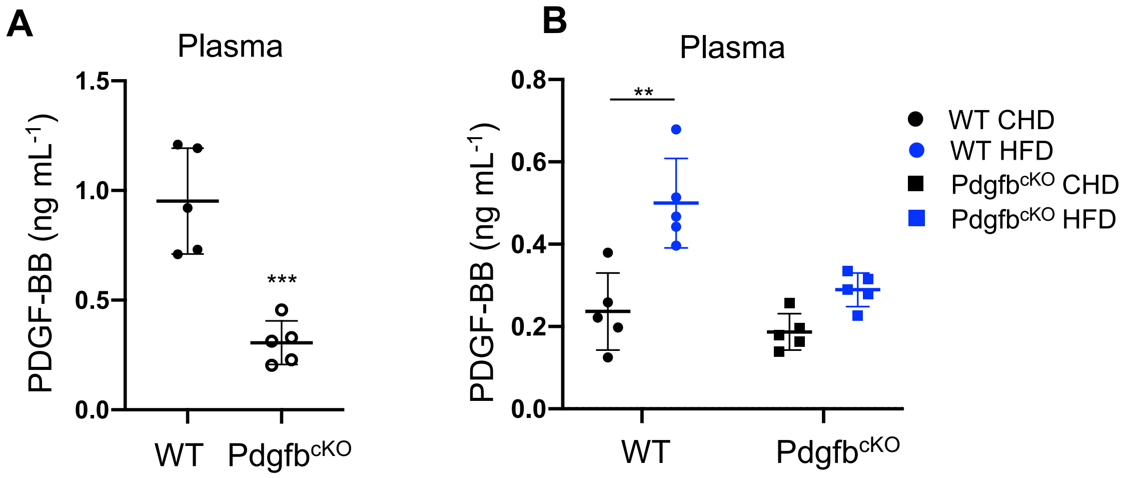

Figure S8. PDGF-BB elevation was significant attenuated in aged Pdgfb^{cKO} mice and Pdgfb^{cKO} mice fed with HFD. ELISA analysis of plasma PDGF-BB concentration in 18-month-old Pdgfb^{cKO} mice and WT littermates (**A**). Pdgfb^{cKO} mice and WT littermates were fed HFD or CHD for 4 months, starting from 3 months of age. ELISA analysis of serum PDGF-BB concentration (**B**). n=5, Data are shown as the mean ± SD, $^{**}p<0.01$, $^{***}p<0.001$, as determined by unpaired two-tailed Student's *t* test.

Figure S9

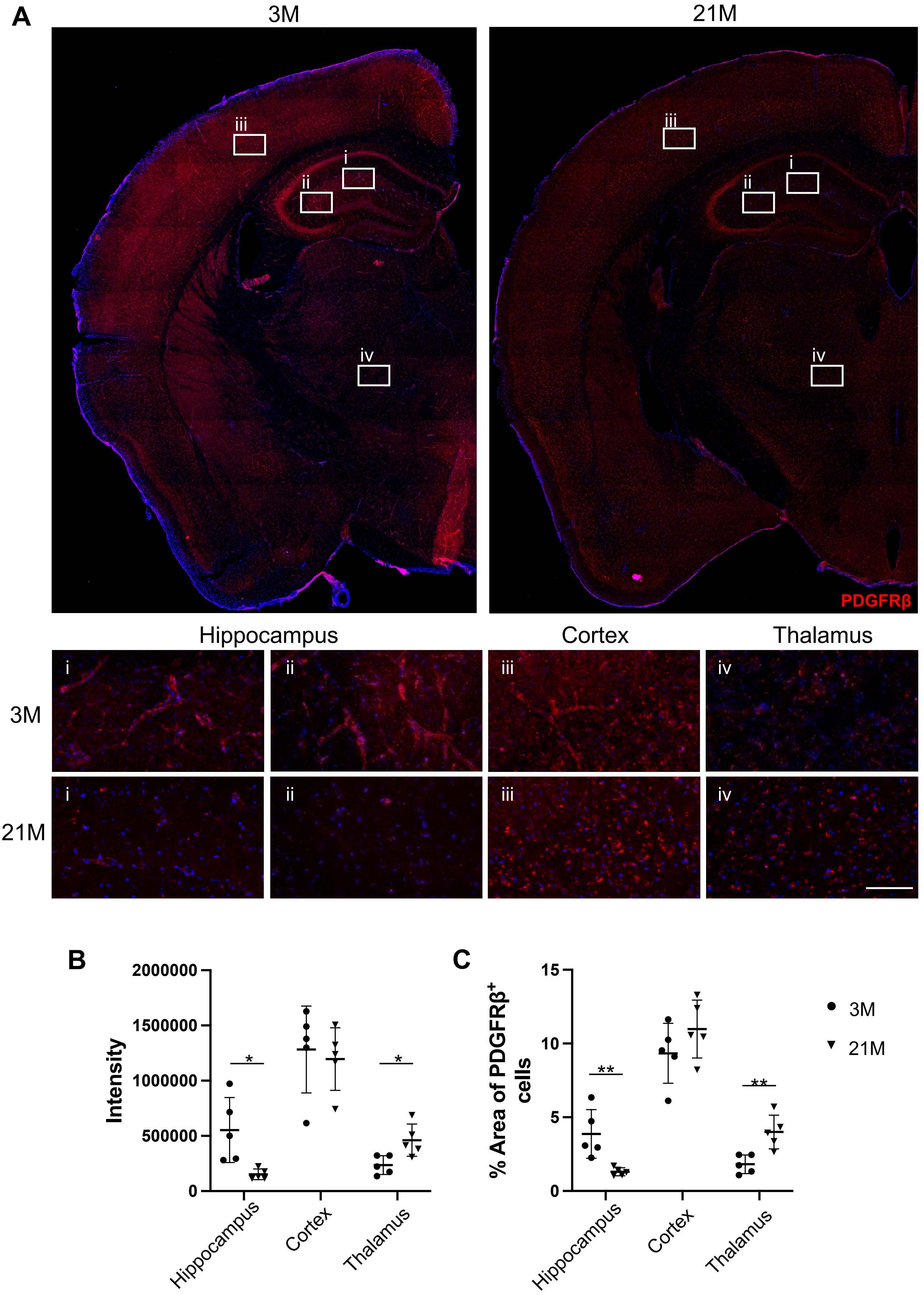
A
3M
21M
iii
i
ii
iv
PDGFRβ
Hippocampus
Cortex
Thalamus
3M
21M
i
ii
iii
iv
B
Intensity
2000000
1500000
1000000
500000
0
*
*
Hippocampus
Cortex
Thalamus
C
% Area of PDGFRβ+ cells
15
10
5
0
**
**
Hippocampus
Cortex
Thalamus
3M
21M

Figure S9. Loss of PDGFRβ expression in the brain of aged WT mice. Representative PDGFRβ immunofluorescence of whole brain images (upper) and enlarged images of different regions (lower) from 3-month-old and 21-month-old WT mice (**A**). DAPI stains nuclei blue. Scale bar, 100 μm. Quantification of intensity (**B**) and % area of PDGFRβ$^+$ cells **(C)** in hippocampus, cortex, thalamus area. n=5. Data are shown as the mean ± SD, *p<0.05, **p<0.01, as determined by unpaired two-tailed Student's *t* test.

Figure S10

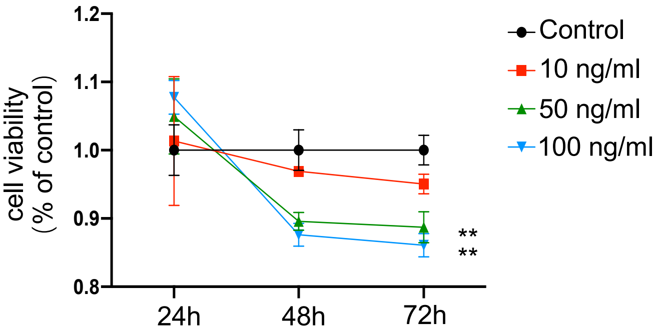

Figure S10. High concentration of PDGF-BB decreased cell viability. Primary human brain pericytes were treated with different dosages of recombinant human PDGF-BB (rh-PDGF-BB) for 24, 48 or 72 hours, cell viability was measure by MTT assay. n=3. Data are shown as the mean ± SD, **p<0.01, as determined by One-way ANOVA.

Figure S11

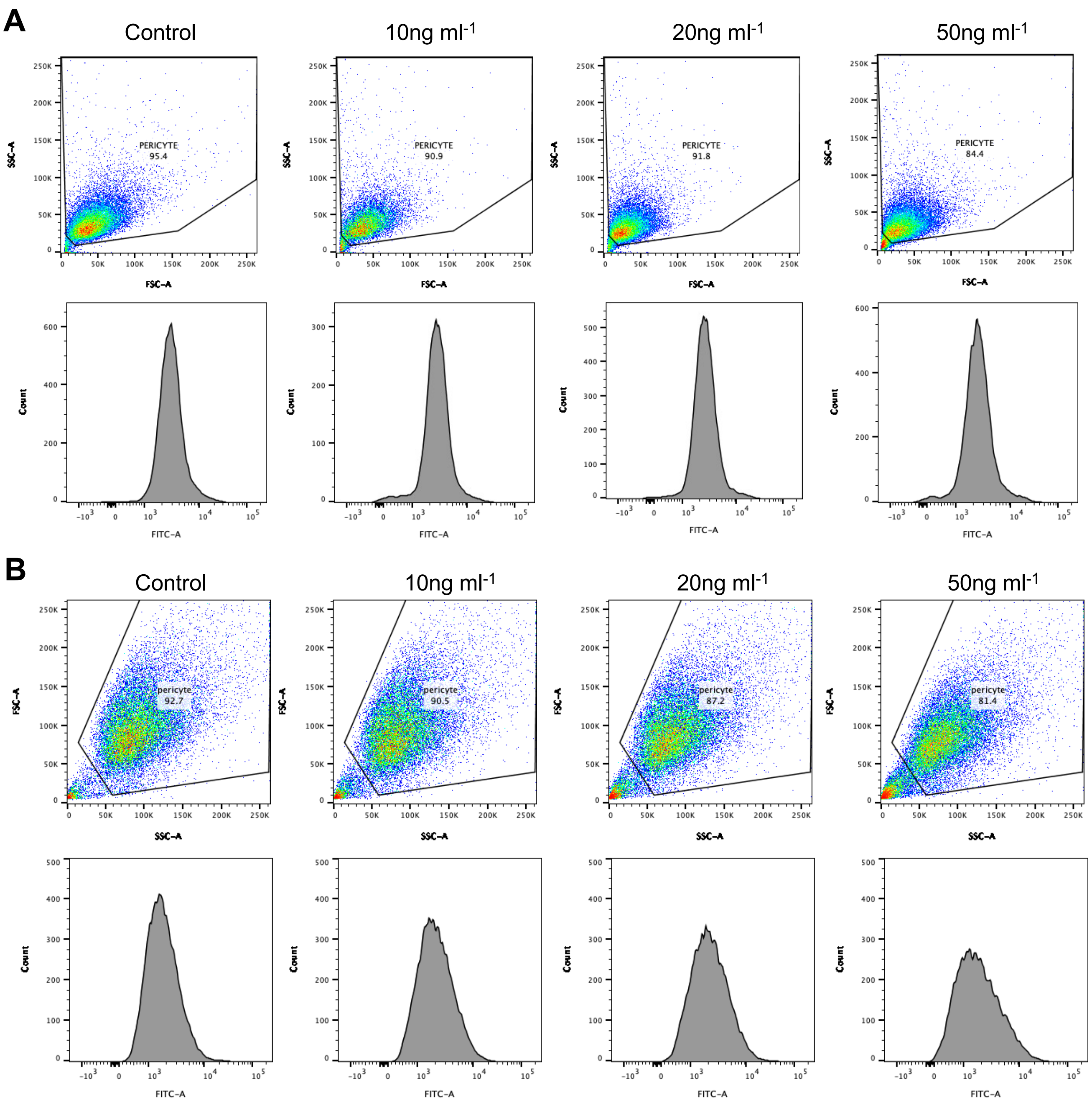

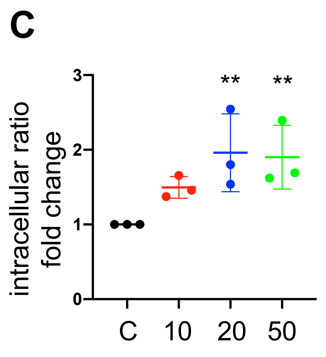

Figure S11. Short-term PDGF-BB treatment exhibit different mechanism in PDGFRβ downregulation. Primary human brain pericytes were treated with different dosages of recombinant human PDGF-BB (rh-PDGF-BB) for 24 hours. Total-PDGFRβ **(A)** and cell surface-PDGFRβ **(B)** were measured by flow cytometry. **(C)** Quantification of intracellular ratio fold change**.** n=3. Data are shown as the mean ± SD, **p<0.01, as determined by One-way ANOVA.

Figure S12

A

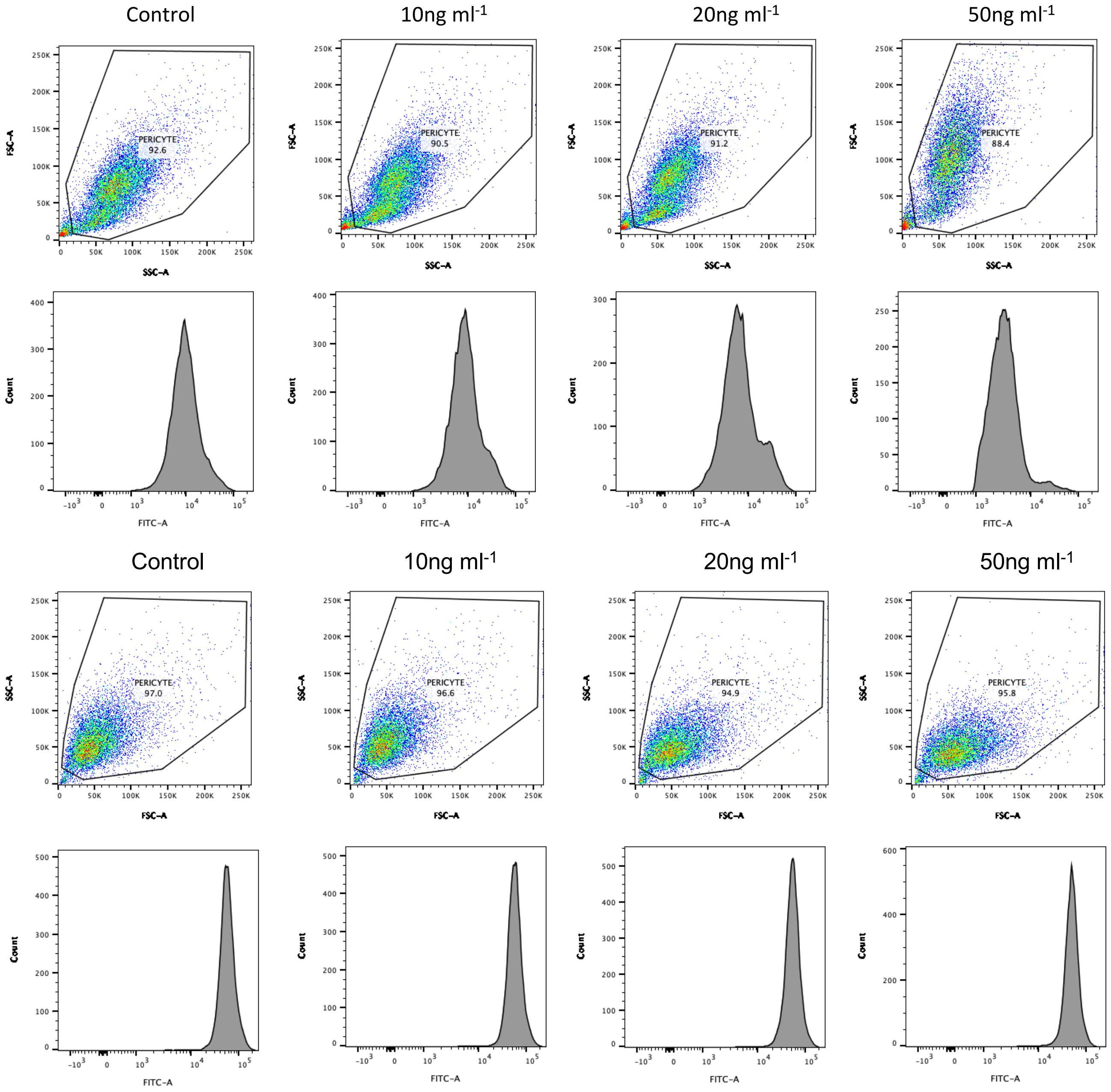

C

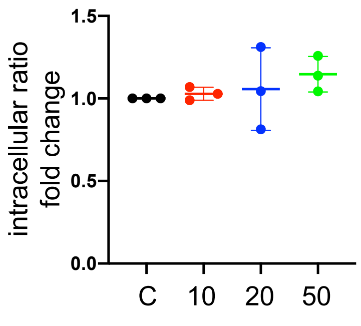

Figure S12. Long-term PDGF-BB treatment exhibit non-internalization in PDGFRβ downregulation. Primary human brain pericytes were treated with different dosages of recombinant human PDGF-BB (rh-PDGF-BB) for 72 hours. Total-PDGFRβ **(A)** and cell surface-PDGFRβ **(B)** were measure by flow cytometry. **(C)** Quantification of intracellular ratio fold change**.** n=3. Data are shown as the mean ± SD, as determined by One-way ANOVA.

Figure S13

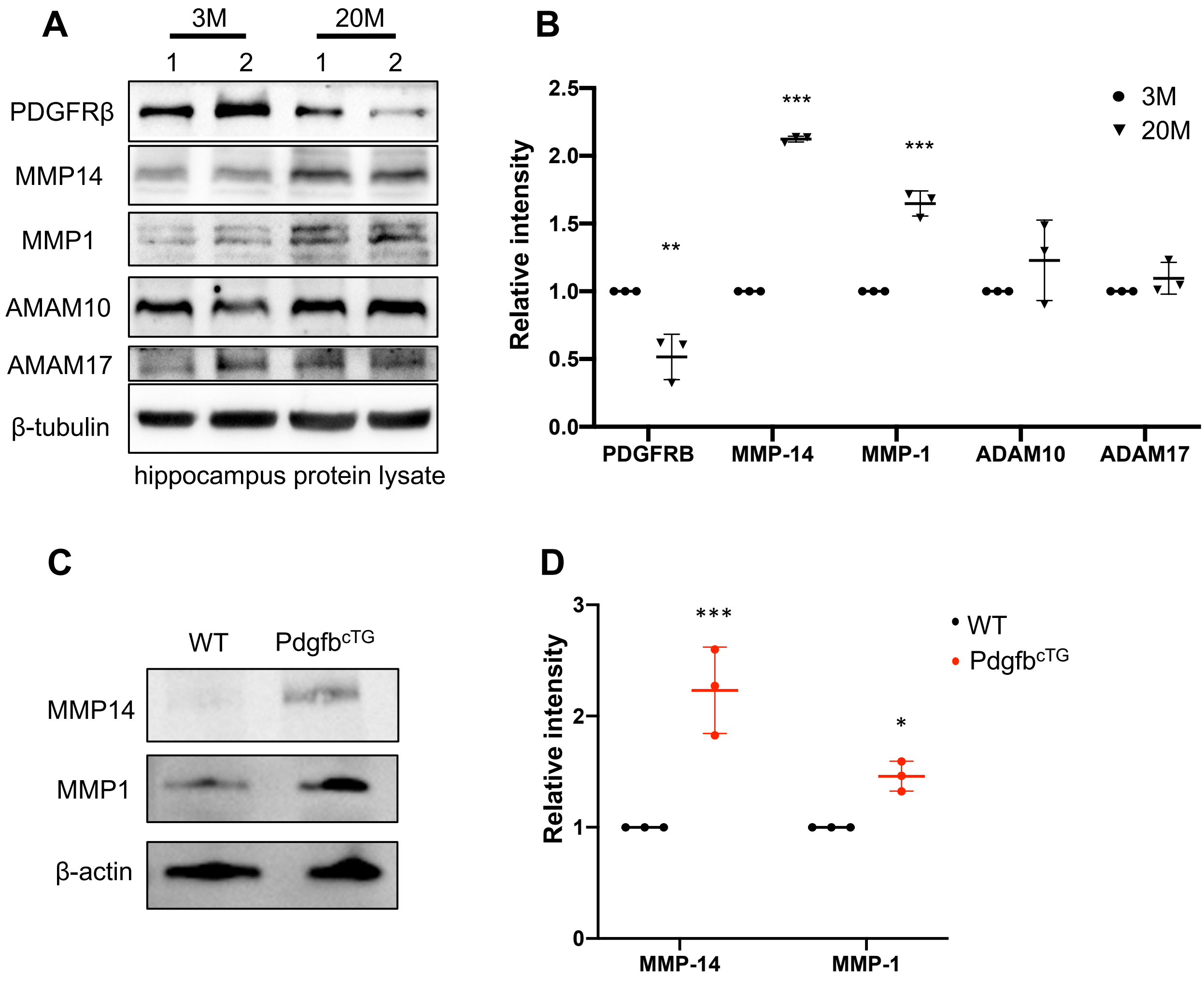

Figure S13. MMP-14 is upregulated in aged and Pdgfb^{cTG} mice. Western blot analysis of PDGFRβ, MMP14, MMP1, ADAM10, ADAM17 protein expression in total hippocampus tissue of 3- and 20-month-old mice **(A)**. Quantification of relative intensity using Image J **(B)**. Western blot analysis of MMP14, MMP1 protein expression in total hippocampus tissue of 6-month-old Pdgfb^{cTG} mice and WT littermates **(C)**. Quantification of relative intensity using Image J (**D**). n=3. Data are shown as the mean ± SD, *p<0.05, ** p<0.01, ***p<0.001, unpaired two-tailed Student's *t* test.

Figure S14

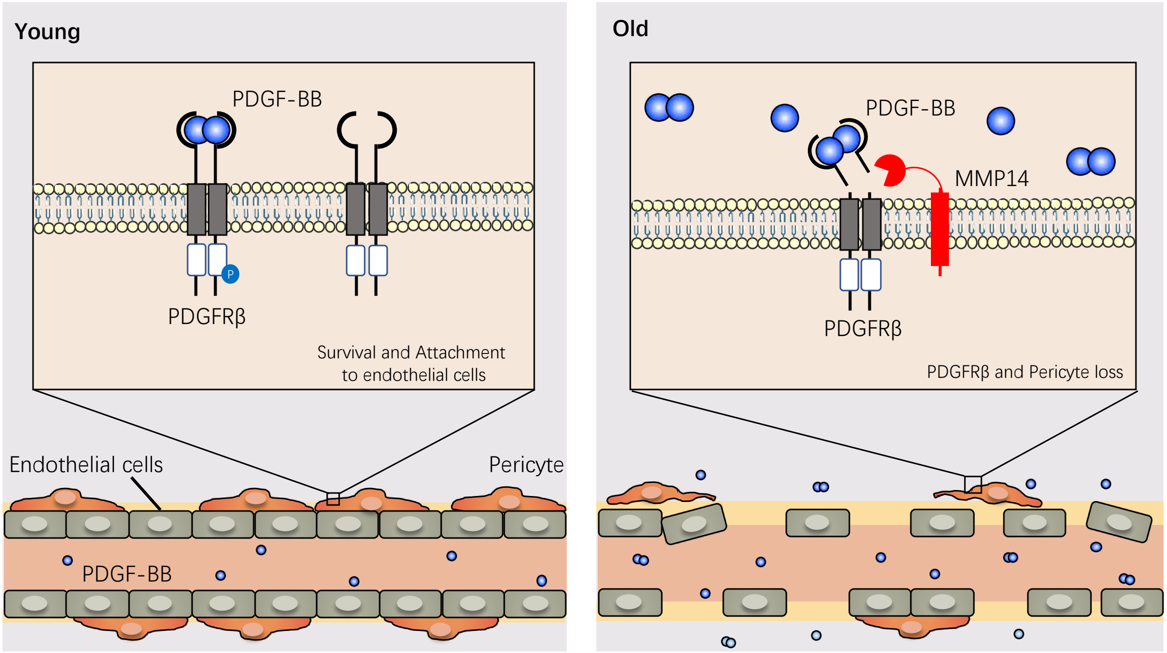

Figure S14. Schematic model of elevated PDGF-BB-induced BBB pericyte loss. In young healthy mice, pericyte survival and attachment to BBB endothelial cells are maintained with normal level of PDGF-BB and active PDGF-BB/PDGFRβ signaling in hippocampus. During aging or under metabolic stress, aberrantly higher concentration of PDGF-BB upregulates MMP14, which cleave the ectodomain of PDGFRβ from pericyte and release of soluble form of PDGFRβ. The deficiency of PDGFRβ signaling activation eventually leads to pericyte loss and BBB disruption.
